# Supplementary material for: Polyacetylenes from the Roots of Swietenia macrophylla King
Source: Molecules. 2019 Apr 2;24(7):1291. doi: 10.3390/molecules24071291 (PMC6480581; doi:10.3390/molecules24071291)
Supplement: Supplementary file 1 [file molecules-24-01291-s001.pdf]

Polyacetylenes from the roots of *Swietenia macrophylla* King

Cheng-Neng Mi [1,2], Hao Wang [1], Hui-Qin Chen [1], Cai-Hong Cai [1], Shao-Peng Li [2], Wen-Li Mei [1,*] and Hao-Fu Dai [1,*]

[1] Key Laboratory of Biology and Genetic Resources of Tropical Crops, Ministry of Agriculture, Institute of Tropical Bioscience and Biotechnology, Chinese Academy of Tropical Agricultural Sciences, 571101, Haikou, PR China; michengnengnpc@126.com (C.-N.M.); wanghao@itbb.or.cn (H.W.); chenhuiqin@itbb.or.cn (H.-Q.C.); caicaihong@itbb.or.cn (C.-H.C.); meiwenli@itbb.or.cn (W.-L.M.); daihaofu@itbb.or.cn (H.-F.D.)

[2] Institute of Tropical Agriculture and Forestry, Hainan University, Haikou 570228, PR China; lisp555@126.com (S.-P.L.)

* Correspondence: meiwenli@itbb.org.cn (W.-L.M.); daihaofu@itbb.org.cn (H.-F.D.); Tel.: +86-898-6698-7529 (W.-L.M.); +86-898-6696-1869 (H.-F.D.)

Table of Content

Figure S1. ^{1}H NMR spectrum (500 MHz) of compound **1** in $CDCl_3$. 3
Figure S2. ^{13}C NMR spectrum (125 MHz) of compound **1** in $CDCl_3$. 4
Figure S3. ^{1}H–^{1}H COSY spectrum of compound **1** in $CDCl_3$. 5
Figure S4. HSQC spectrum of compound **1** in $CDCl_3$. 6
Figure S5. HMBC spectrum of compound **1** in $CDCl_3$. 7
Figure S6. ROESY spectrum of compound **1** in $CDCl_3$. 8
Figure S7. HRESI(+)MS spectrum of compound **1**. 9
Figure S8. ^{1}H NMR spectrum (500 MHz) of compound **2** in $CDCl_3$. 10
Figure S9. ^{13}C NMR spectrum (125 MHz) of compound **2** in $CDCl_3$. 11
Figure S10. ^{1}H–^{1}H COSY spectrum of compound **2** in $CDCl_3$. 12
Figure S11. HSQC spectrum of compound **2** in $CDCl_3$. 13
Figure S12. HMBC spectrum of compound **2** in $CDCl_3$. 14
Figure S13. ROESY spectrum of compound **2** in $CDCl_3$. 15
Figure S14. HRESI(+)MS spectrum of compound **2**. 16
Figure S15. ^{1}H NMR spectrum (500 MHz) of compound **3** in $CDCl_3$. 17
Figure S16. ^{13}C NMR spectrum (125 MHz) of compound **3** in $CDCl_3$. 18
Figure S17. ^{1}H–^{1}H COSY spectrum of compound **3** in $CDCl_3$. 19
Figure S18. HSQC spectrum of compound **3** in $CDCl_3$. 20
Figure S19. HMBC spectrum of compound **3** in $CDCl_3$. 21
Figure S20. ROESY spectrum of compound **3** in $CDCl_3$. 22
Figure S21. HRESI(+)MS spectrum of compound **3**. 23
Figure S22. ^{1}H NMR spectrum (500 MHz) of compound **4** in $CDCl_3$. 24
Figure S23. ^{13}C NMR spectrum (125 MHz) of compound **4** in $CDCl_3$. 25
Figure S24. ^{1}H–^{1}H COSY spectrum of compound **4** in $CDCl_3$. 26
Figure S25. HSQC spectrum of compound **4** in $CDCl_3$. 27
Figure S26. HMBC spectrum of compound **4** in $CDCl_3$. 28
Figure S27. ROESY spectrum of compound **4** in $CDCl_3$. 29
Figure S28. HRESI(+)MS spectrum of compound **4**. 30
Figure S29. ^{1}H NMR spectrum (500 MHz) of compound **5** in $CDCl_3$. 31
Figure S30. ^{13}C NMR spectrum (125 MHz) of compound **5** in $CDCl_3$. 32
Figure S31. ^{1}H–^{1}H COSY spectrum of compound **5** in $CDCl_3$. 33
Figure S32. HSQC spectrum of compound **5** in $CDCl_3$. 34
Figure S33. HMBC spectrum of compound **5** in $CDCl_3$. 35
Figure S34. ROESY spectrum of compound **5** in $CDCl_3$. 36
Figure S35. HRESI(+)MS spectrum of compound **5**. 37

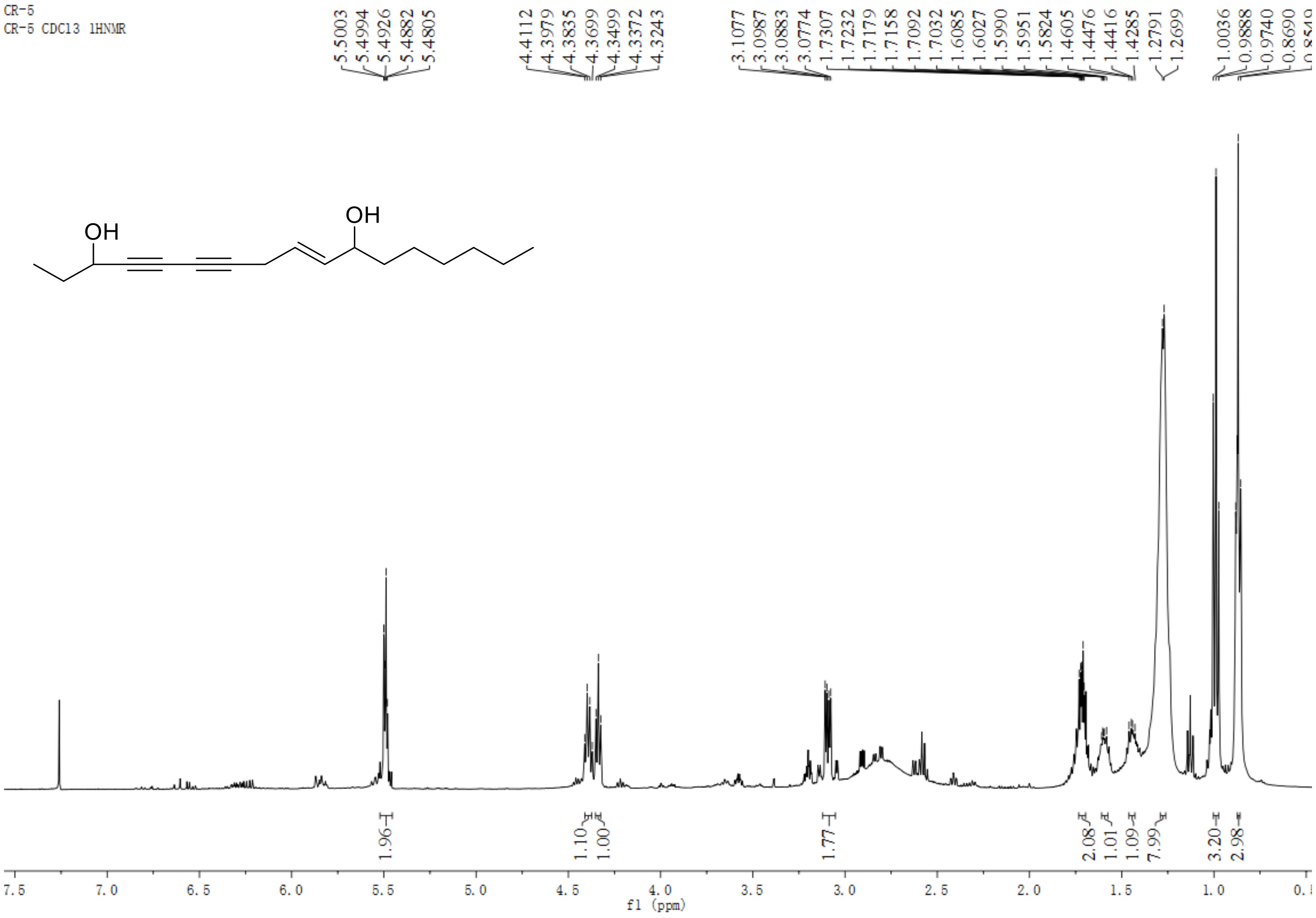

Figure S1. ^{1}H NMR spectrum (500 MHz) of compound **1** in $CDCl_3$.

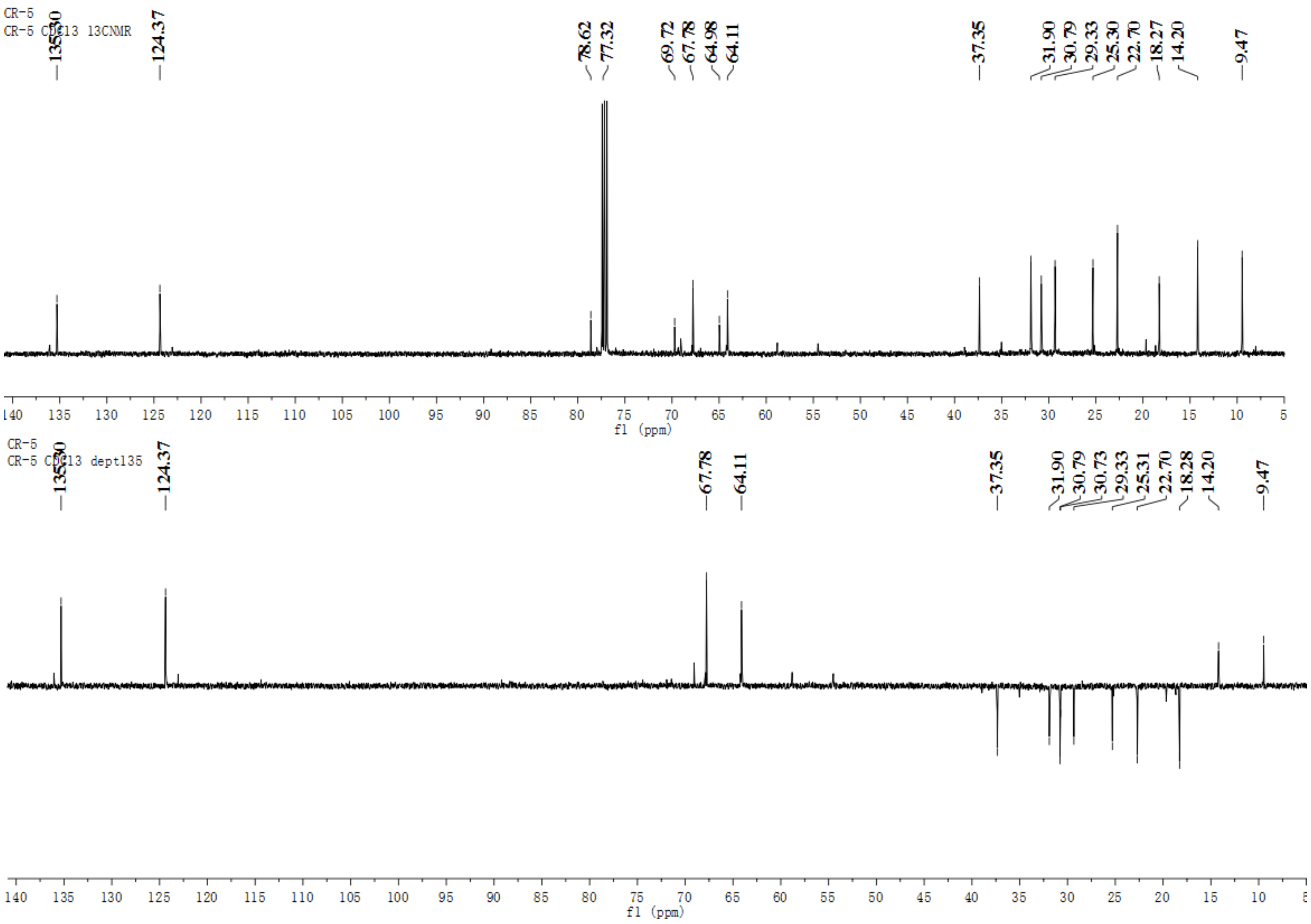

Figure S2. ^{13}C NMR spectrum (125 MHz) of compound **1** in $CDCl_3$.

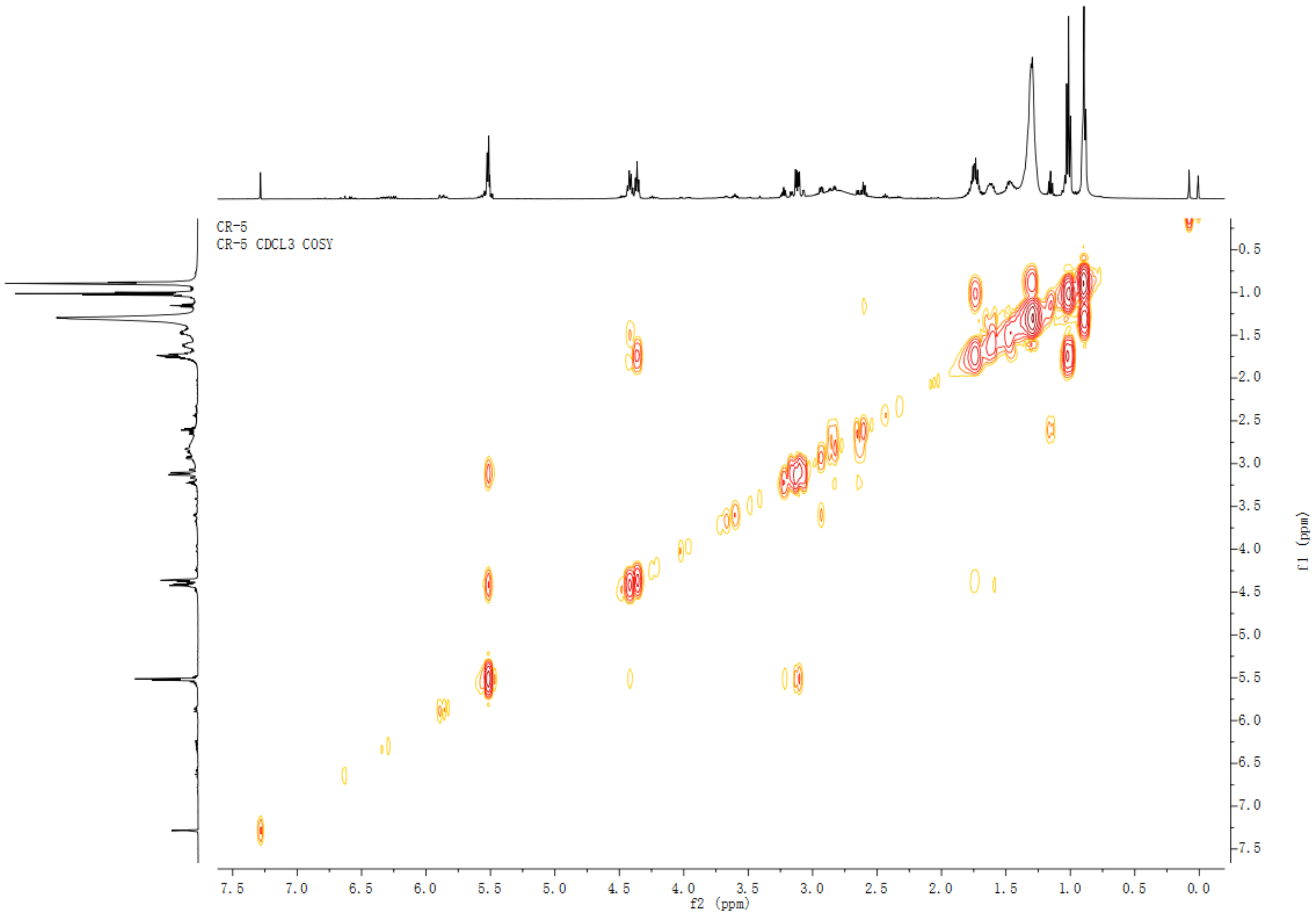

Figure S3. ^{1}H–^{1}H COSY spectrum of compound **1** in $CDCl_3$.

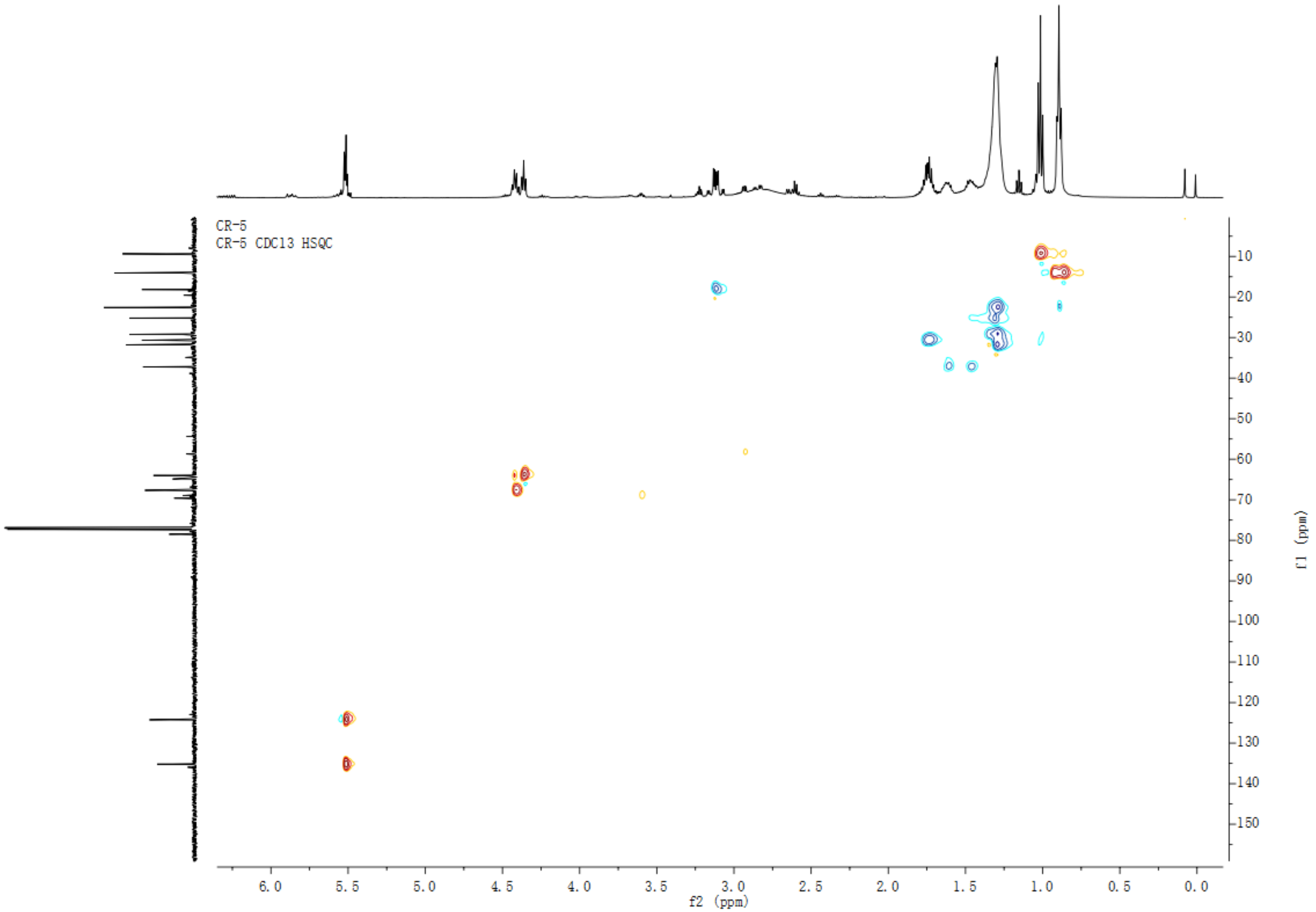

Figure S4. HSQC spectrum of compound **1** in $CDCl_3$.

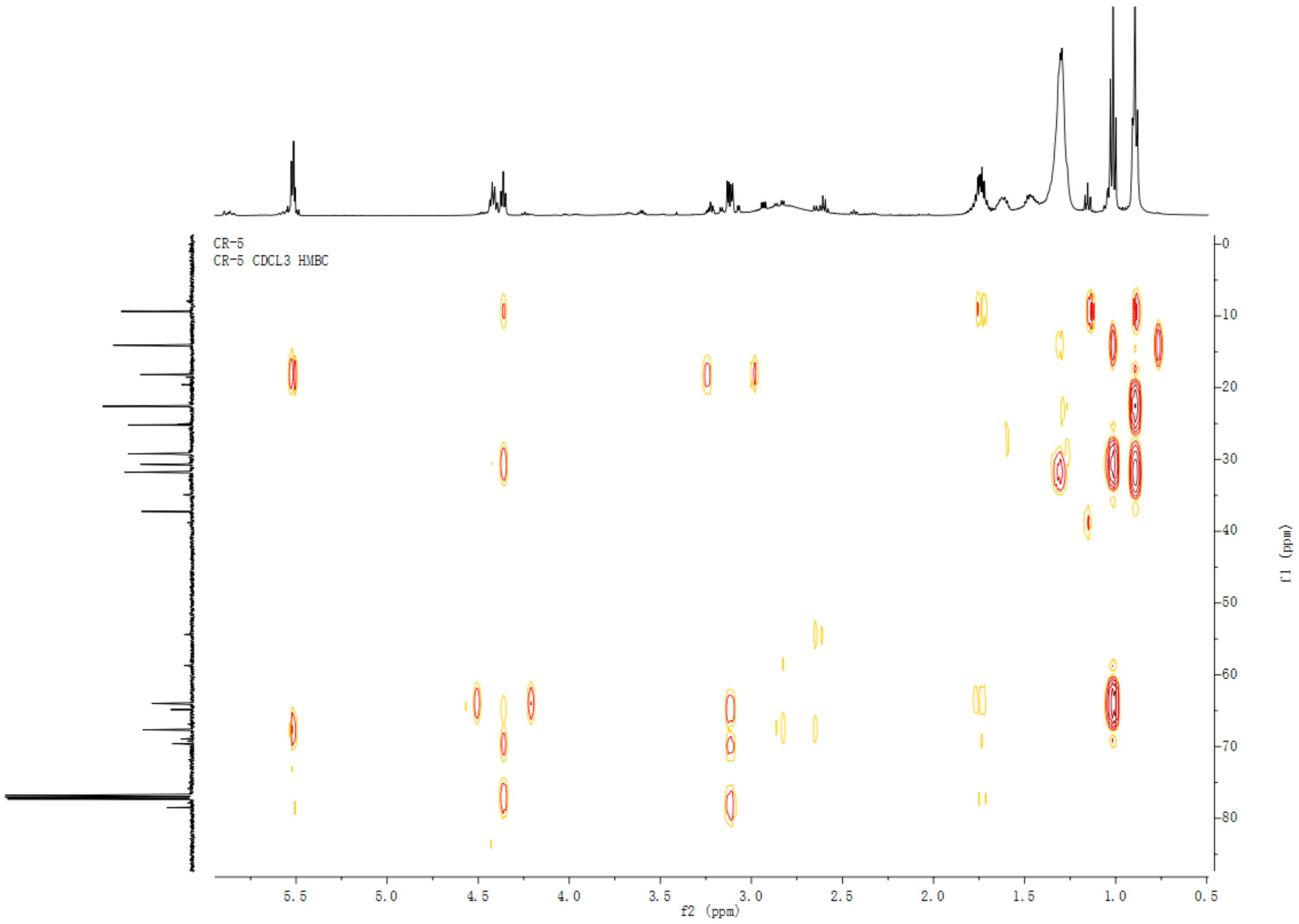

Figure S5. HMBC spectrum of compound **1** in $CDCl_3$.

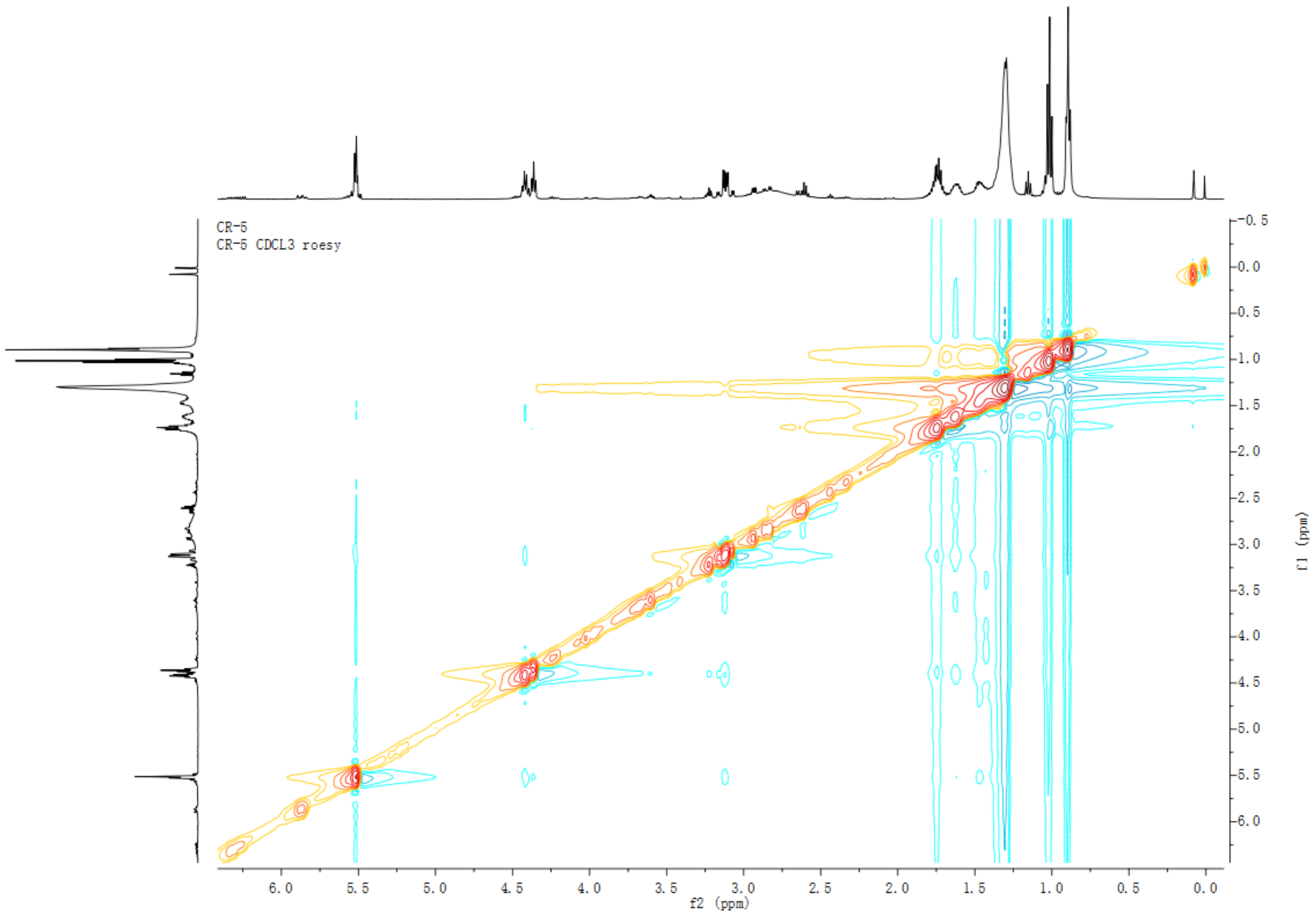

Figure S6. ROESY spectrum of compound **1** in $CDCl_3$.

Qualitative Analysis Report

**Data Filename**	150113ESIA4.d	**Sample Name**	CR-5
**Sample Type**	Sample	**Position**	
**Instrument Name**	Agilent G6230 TOF MS	**User Name**	KIB
**Acq Method**	ESI.m	**Acquired Time**	1/12/2015 1:50:27 PM
**IRM Calibration Status**	Success	**DA Method**	ESI.m
**Comment**			

**Sample Group**		**Info.**
**Acquisition SW Version**	6200 series TOF/6500 series Q-TOF B.05.01 (B5125.2)	

User Spectra

Fragmentor Voltage	Collision Energy	Ionization Mode
240	0	ESI

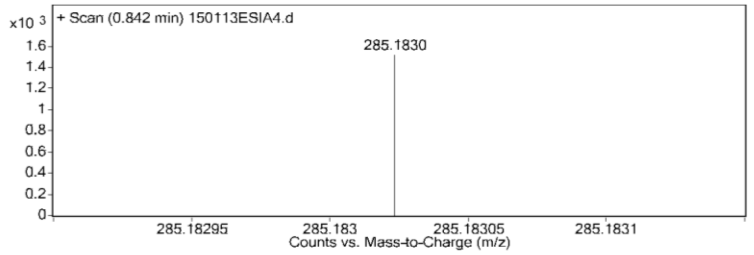

Peak List

m/z	z	Abund
105.0458		4378.09
121.0509	1	8292.57
193.0494	1	2694.31
274.2738	1	3758.84
318.3002	1	4489.7
512.5038	1	2579.33
540.5347	1	3205.15
922.0098	1	32392.58
923.0125	1	5390.13
1821.9496	1	3029.58

Formula Calculator Element Limits

Element	Min	Max
C	0	200
H	0	400
O	0	8
Na	1	1

Formula Calculator Results

Formula	CalculatedMass	Mz	Diff.(mDa)	Diff. (ppm)	DBE
C17 H26 Na O2	285.1831	285.1830	0.0	0.2	4.5

--- End Of Report ---

Figure S7. HRESI(+)MS spectrum of compound **1**.

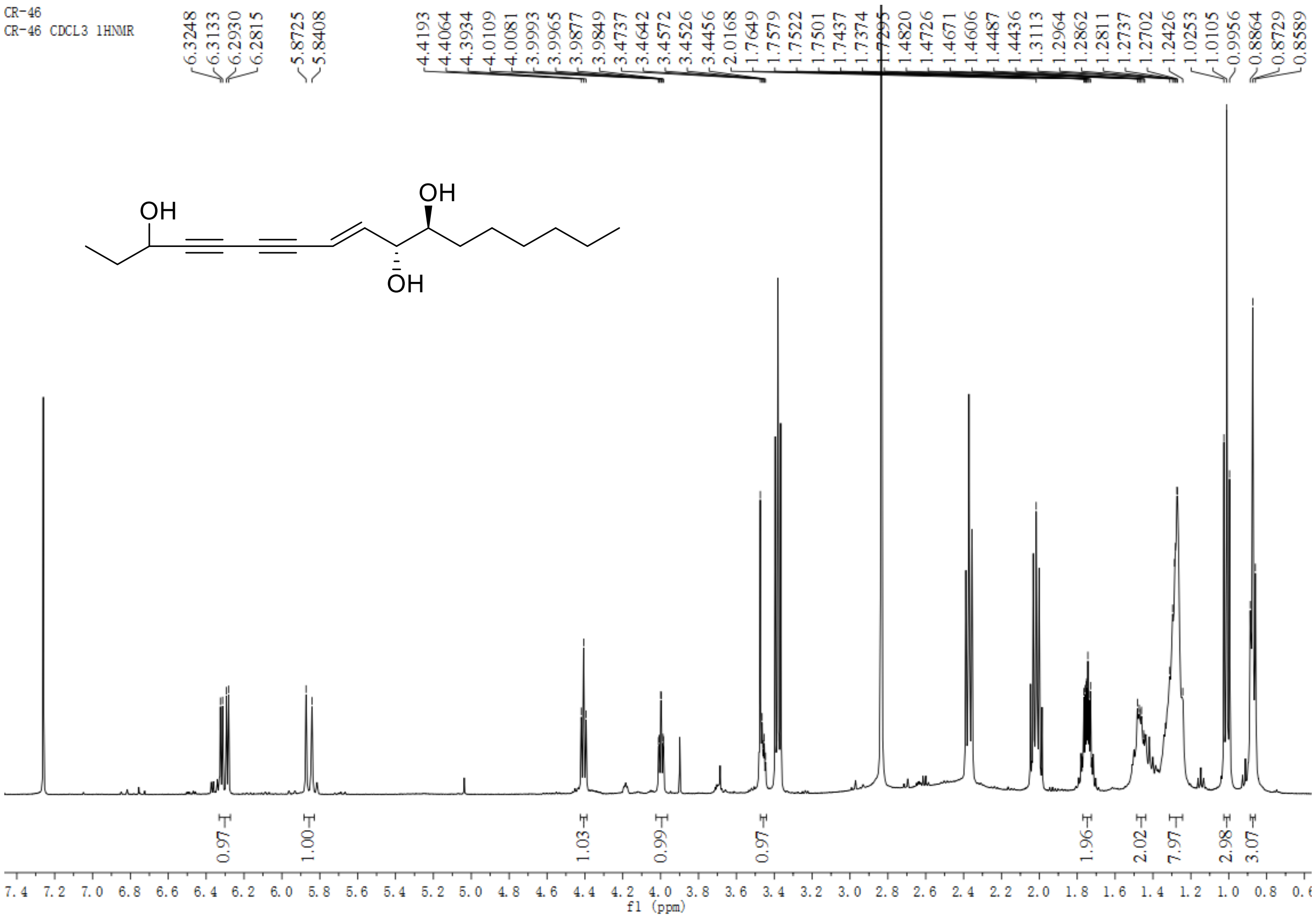

Figure S8. ^{1}H NMR spectrum (500 MHz) of compound **2** in $CDCl_3$.

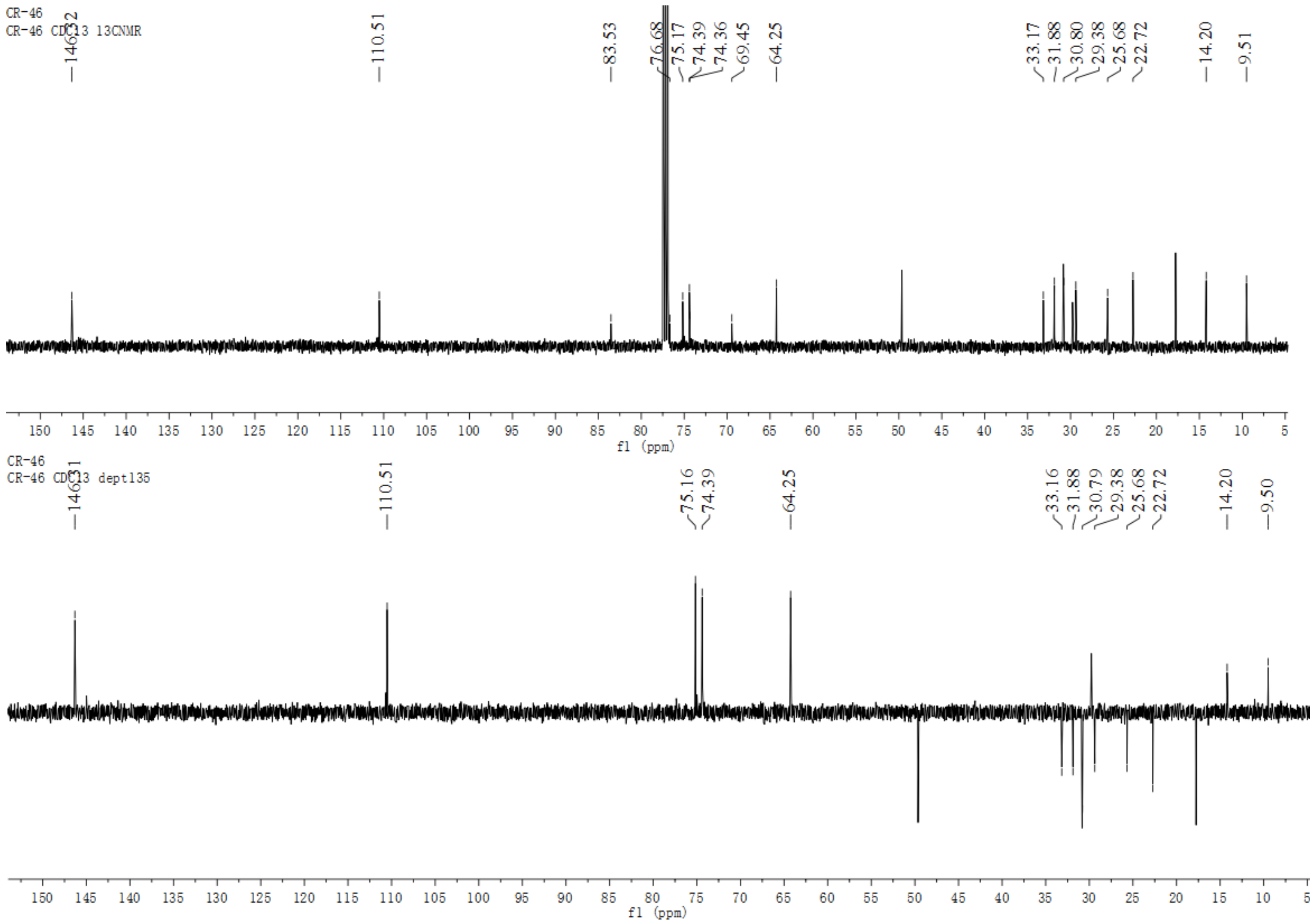

Figure S9. ^{13}C NMR spectrum (125 MHz) of compound **2** in $CDCl_3$.

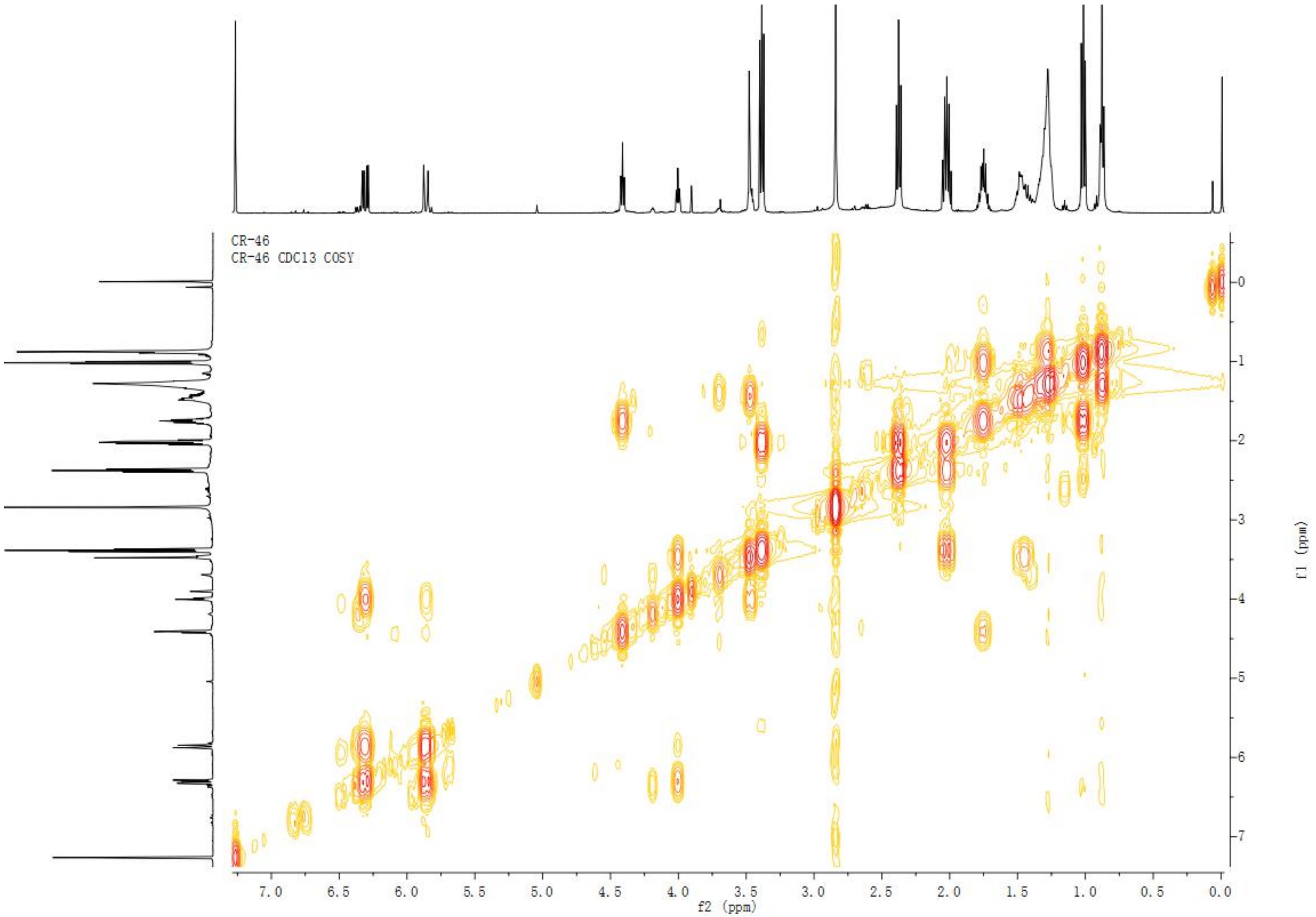

Figure S10. ^{1}H–^{1}H COSY spectrum of compound **2** in $CDCl_3$.

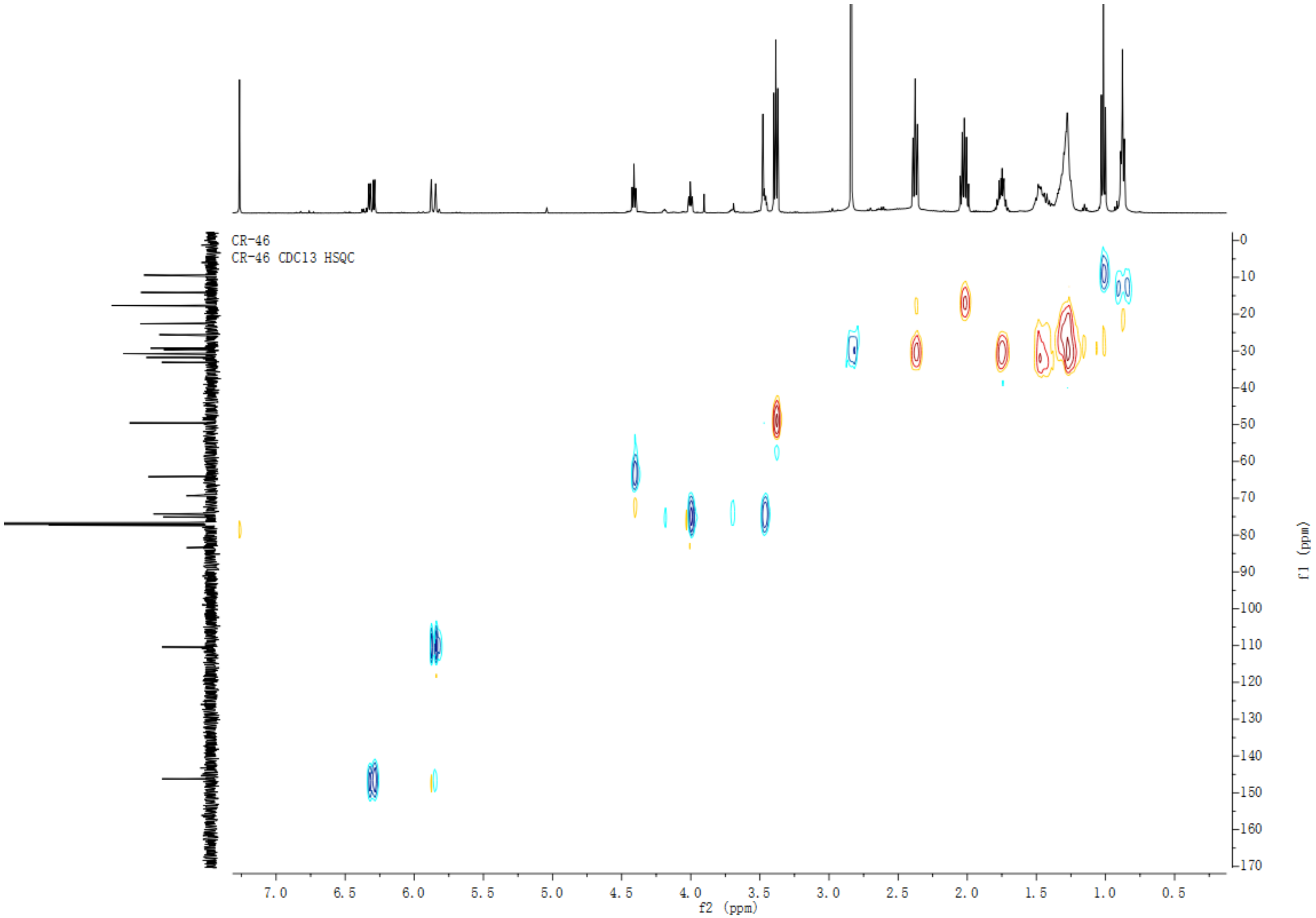

Figure S11. HSQC spectrum of compound **2** in $CDCl_3$.

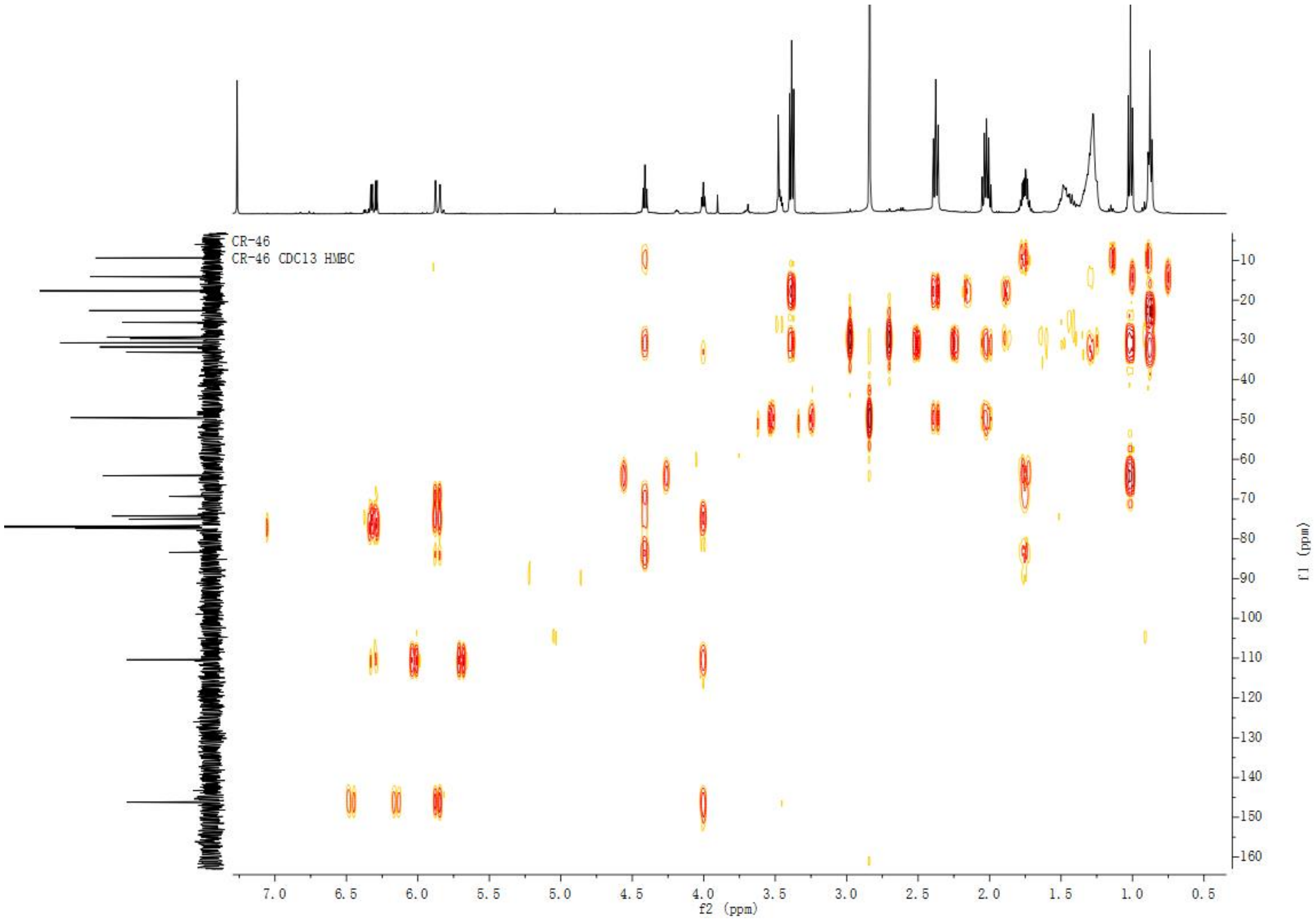

Figure S12. HMBC spectrum of compound **2** in $CDCl_3$.

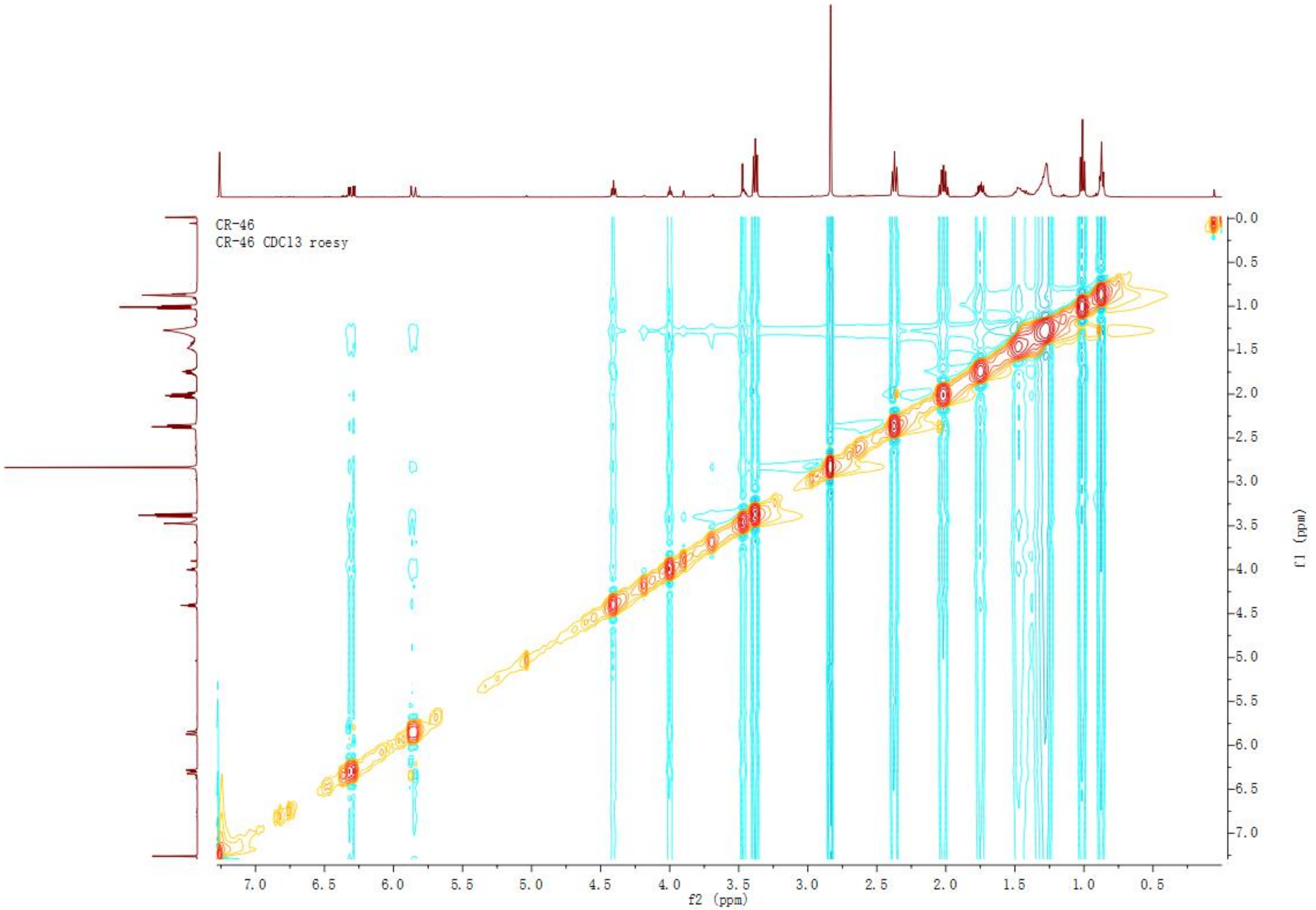

Figure S13. ROESY spectrum of compound **2** in $CDCl_3$.

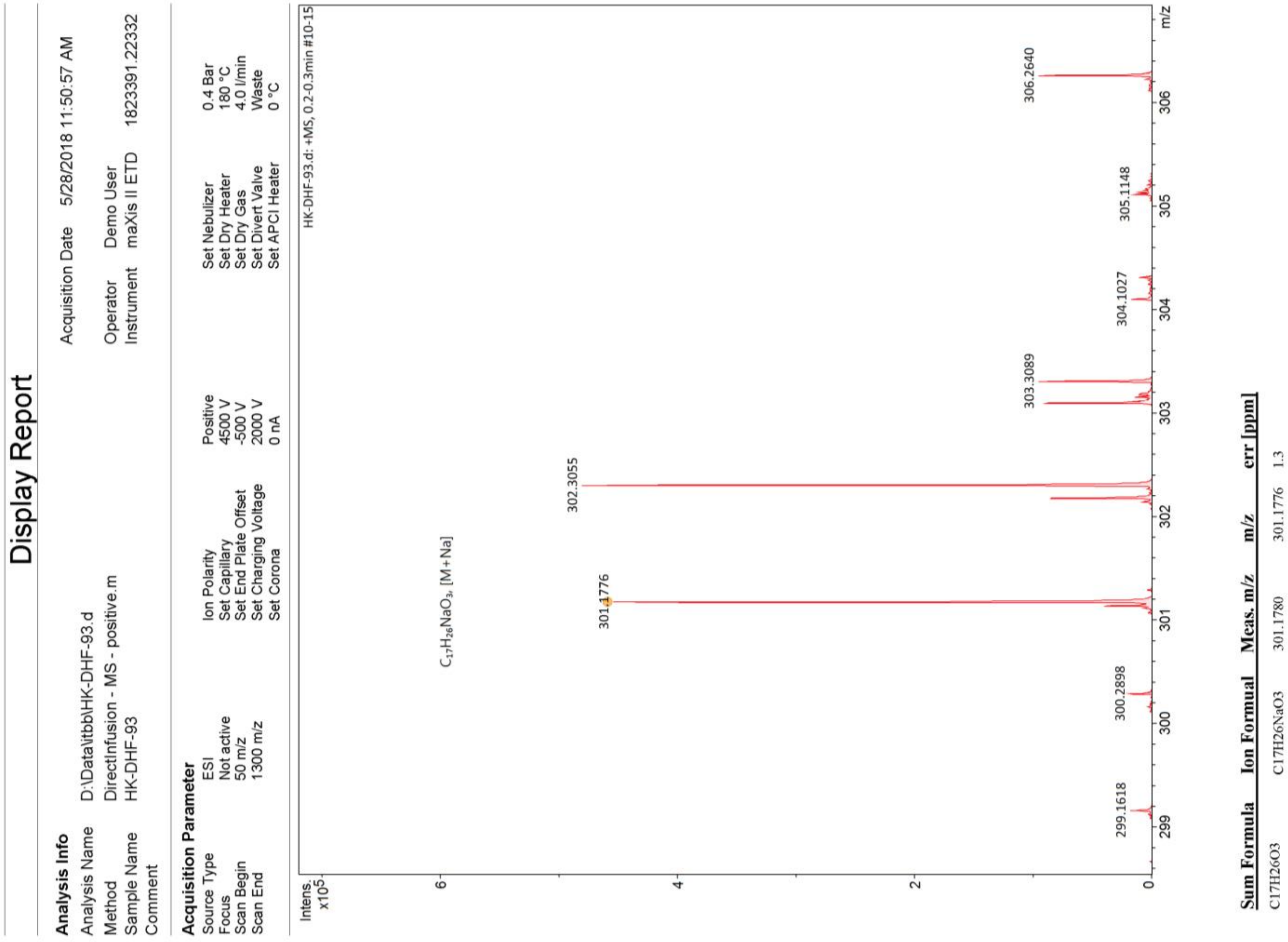

Figure S14. HRESI(+)MS spectrum of compound **2**.

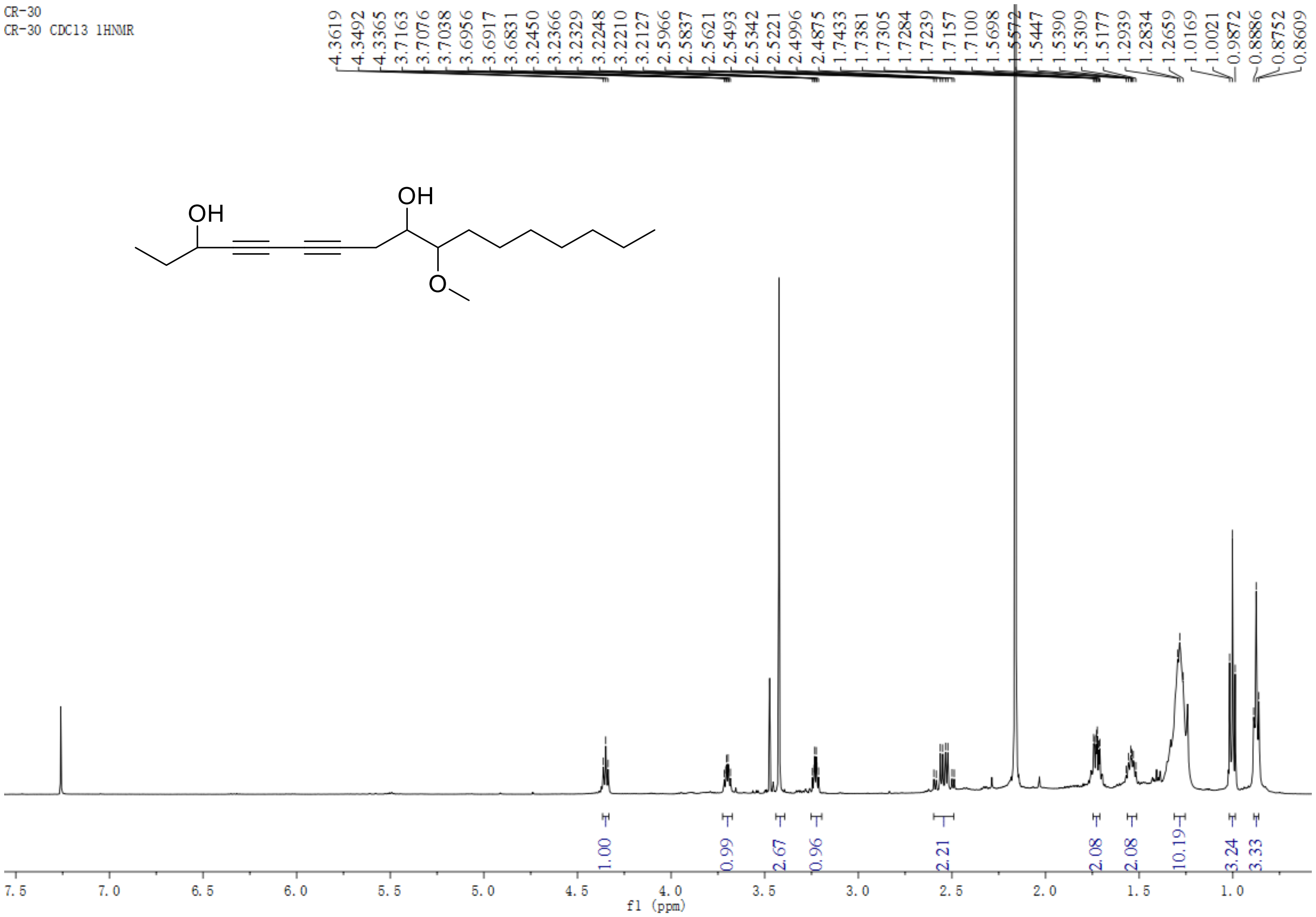

Figure S15. ^{1}H NMR spectrum (500 MHz) of compound **3** in $CDCl_3$.

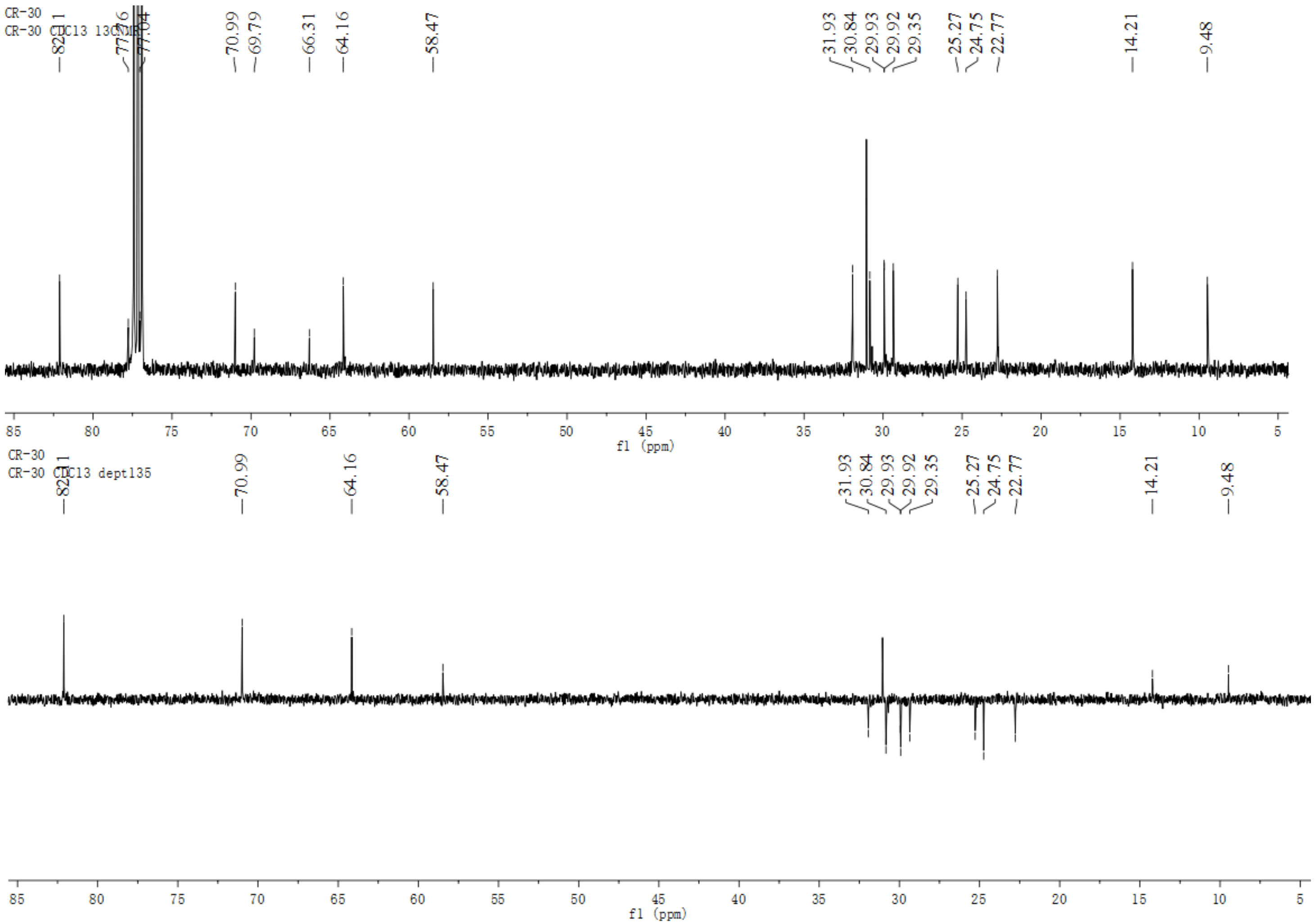

Figure S16. ^{13}C NMR spectrum (125 MHz) of compound **3** in $CDCl_3$.

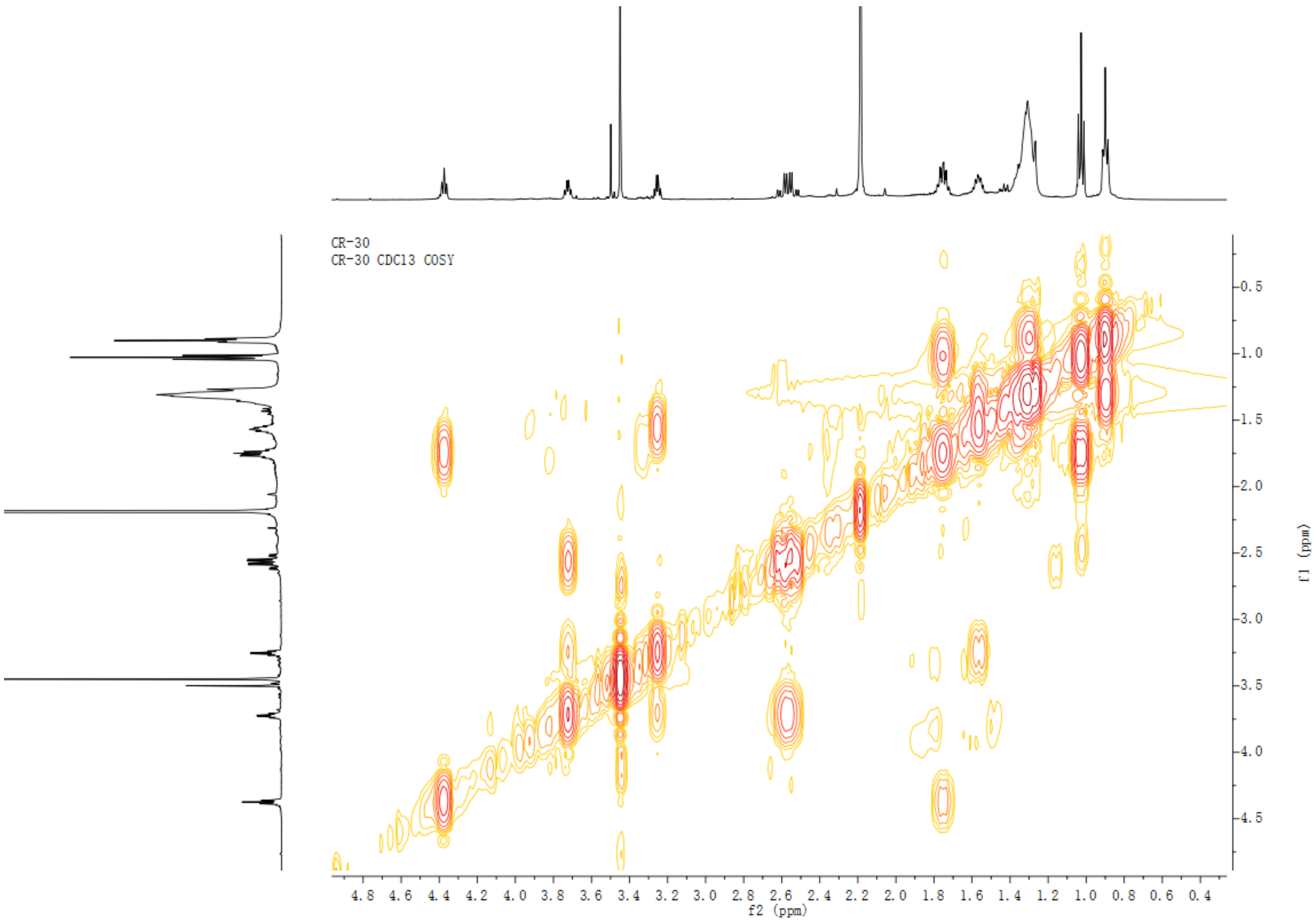

Figure S17. ^1H–^1H COSY spectrum of compound **3** in $CDCl_3$.

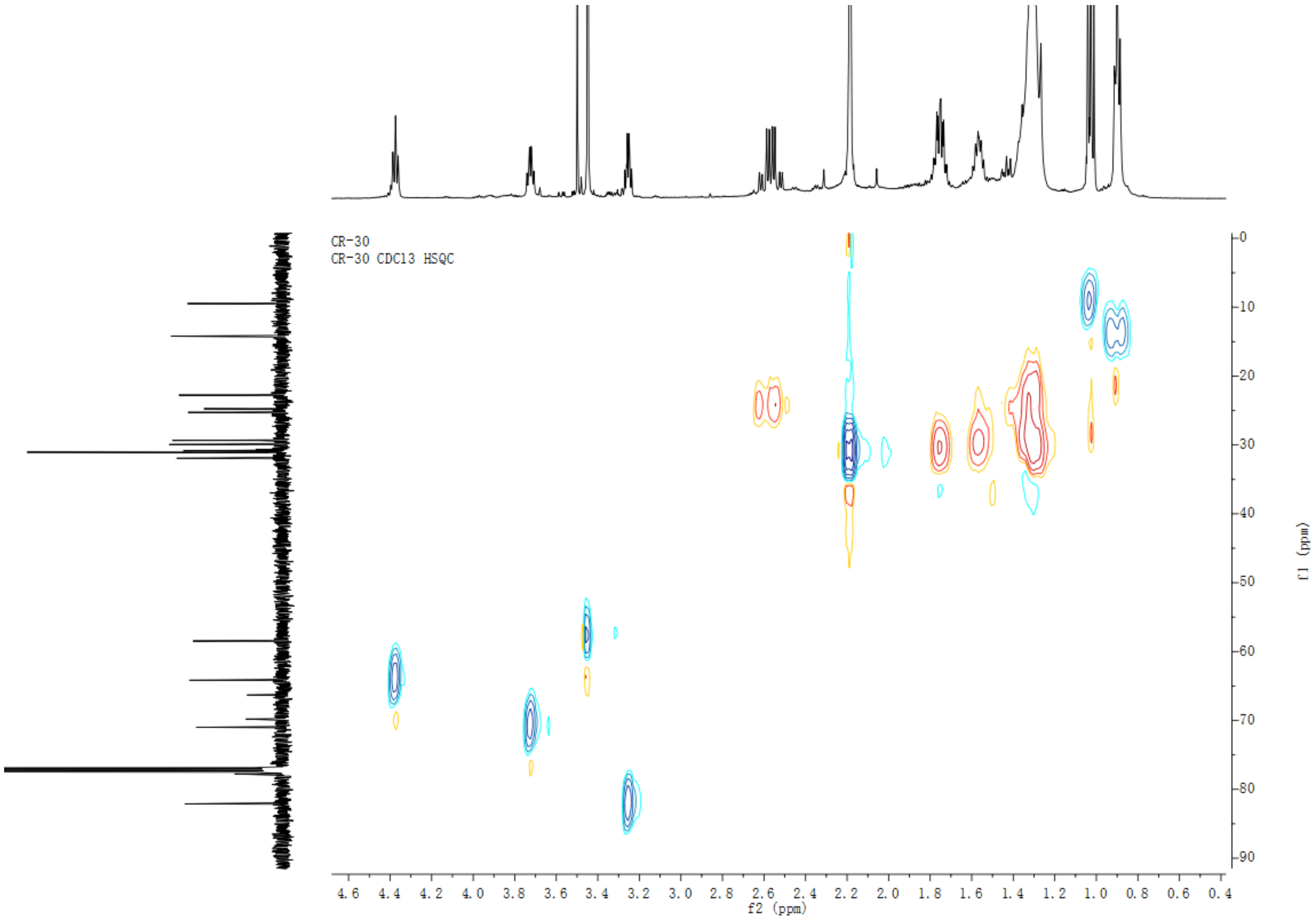

Figure S18. HSQC spectrum of compound **3** in $CDCl_3$.

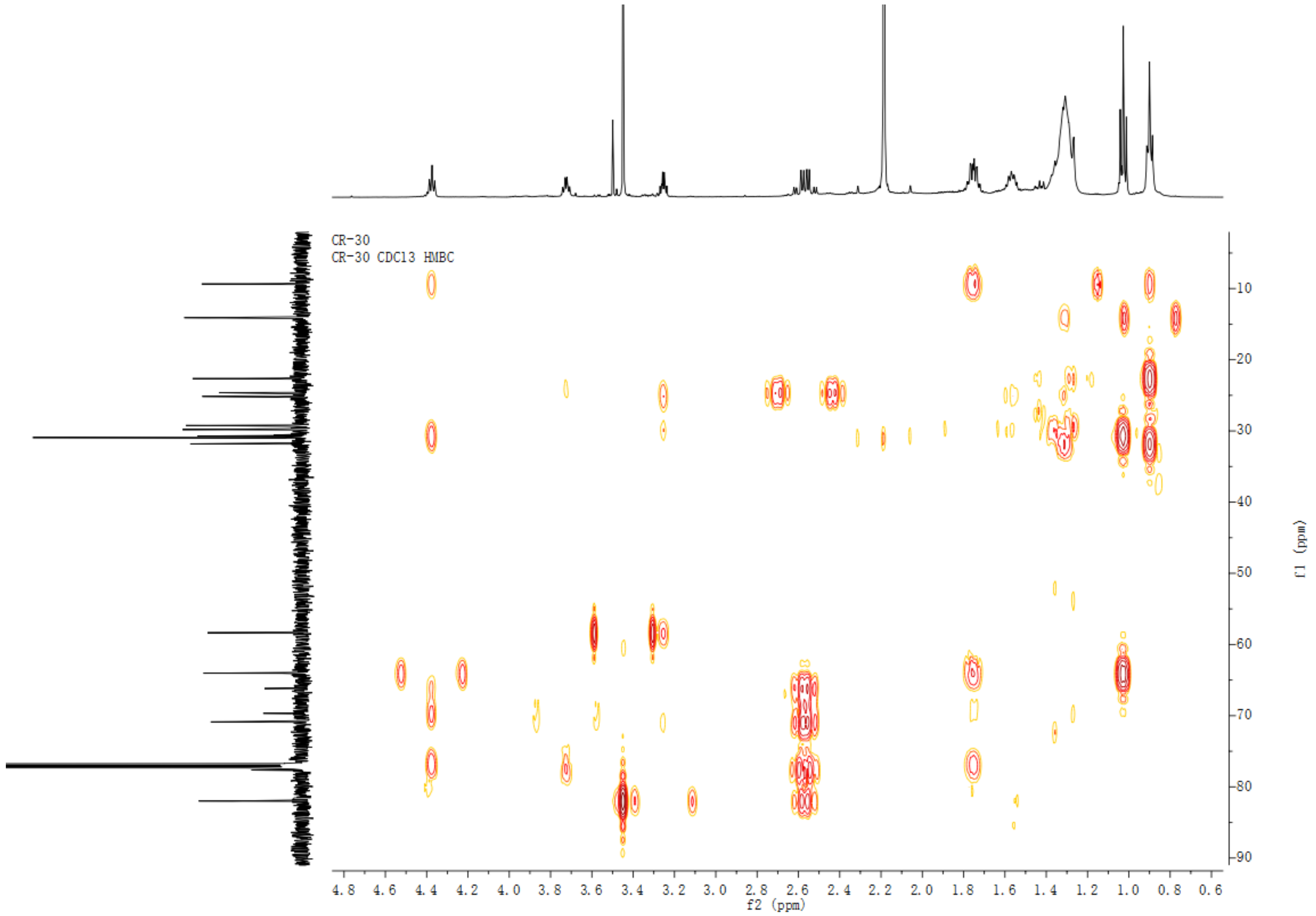

Figure S19. HMBC spectrum of compound **3** in $CDCl_3$.

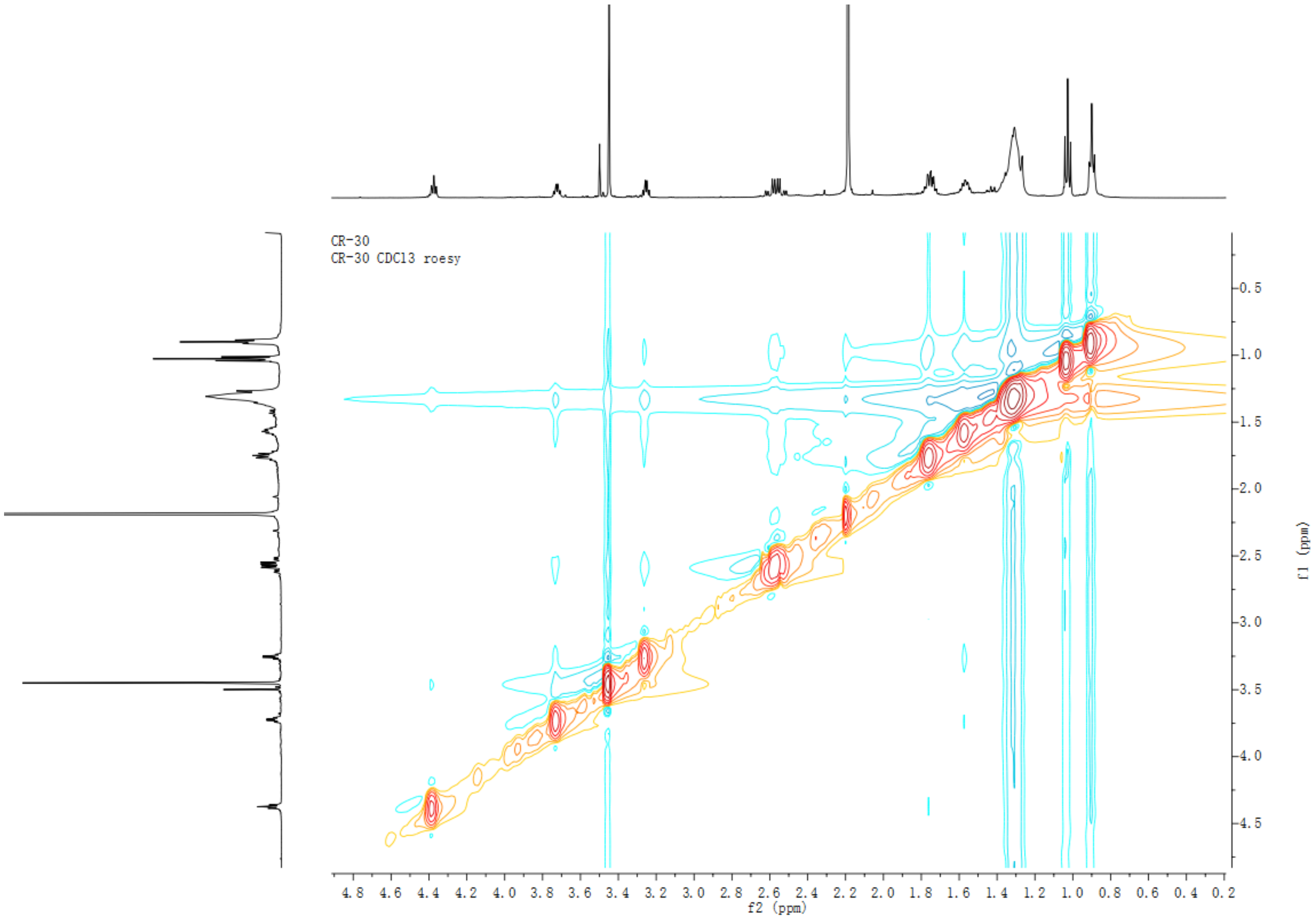

Figure S20. ROESY spectrum of compound **3** in $CDCl_3$.

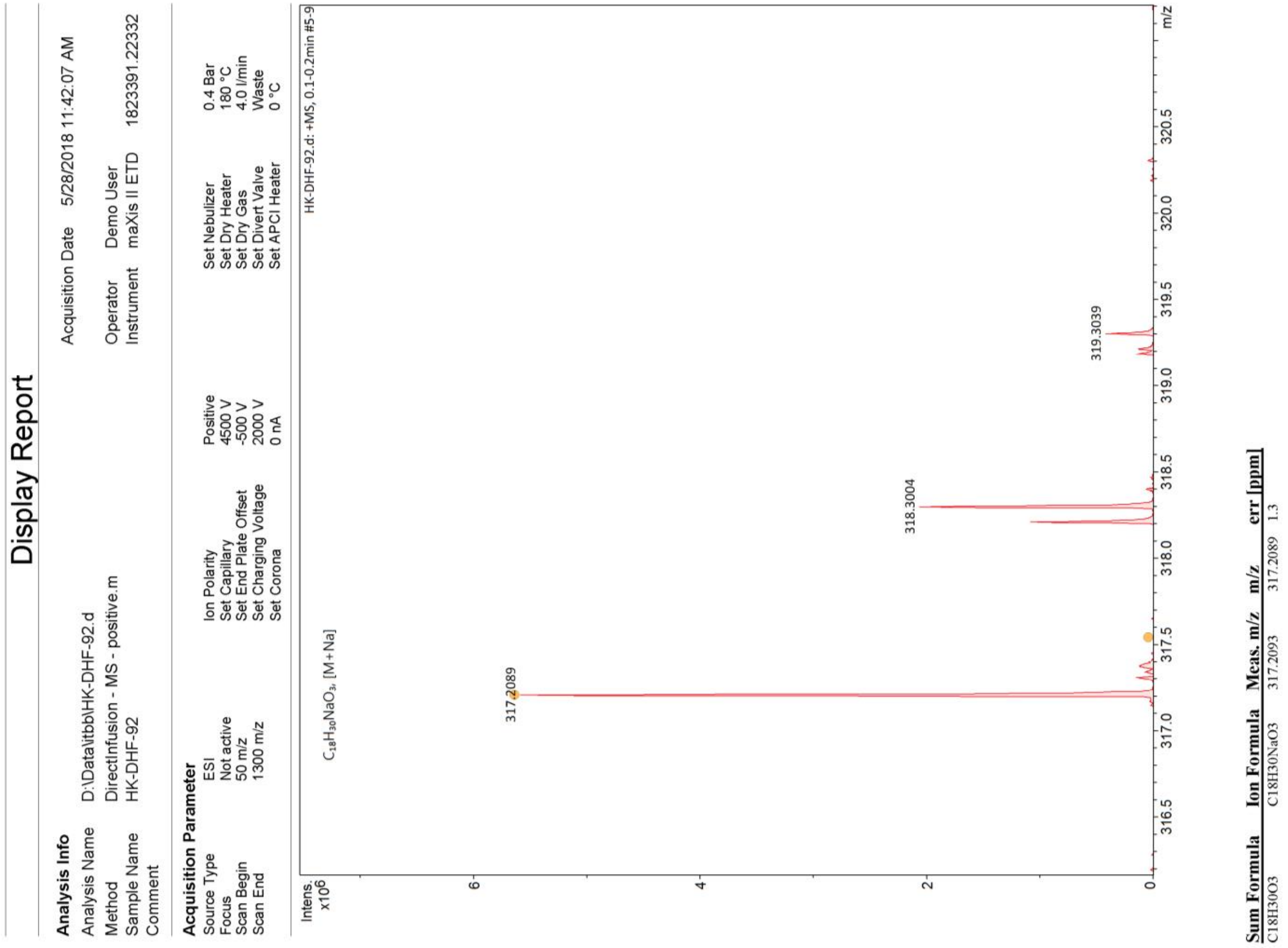

Figure S21. HRESI(+)MS spectrum of compound **3**.

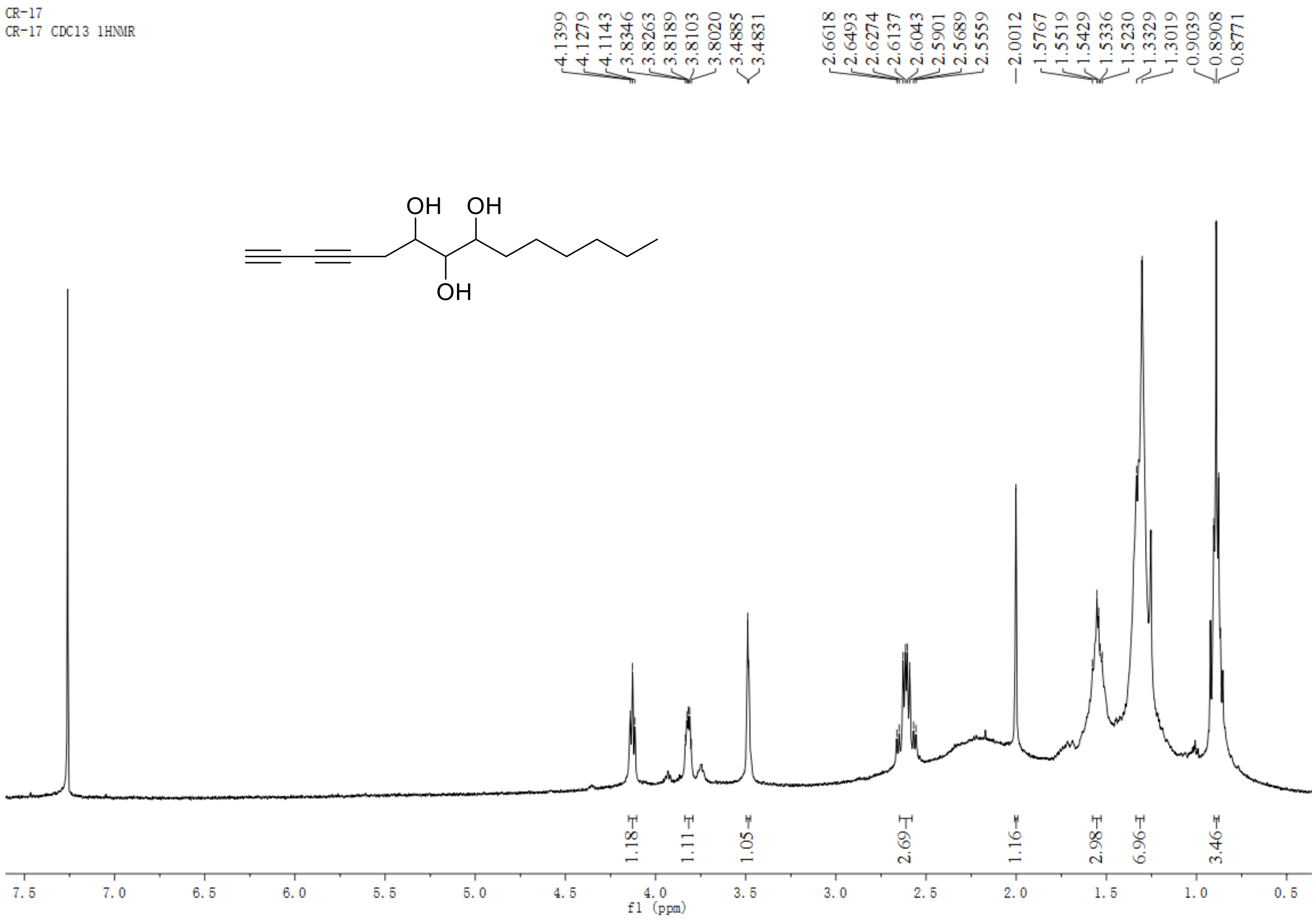

Figure S22. ^{1}H NMR spectrum (500 MHz) of compound **4** in $CDCl_3$.

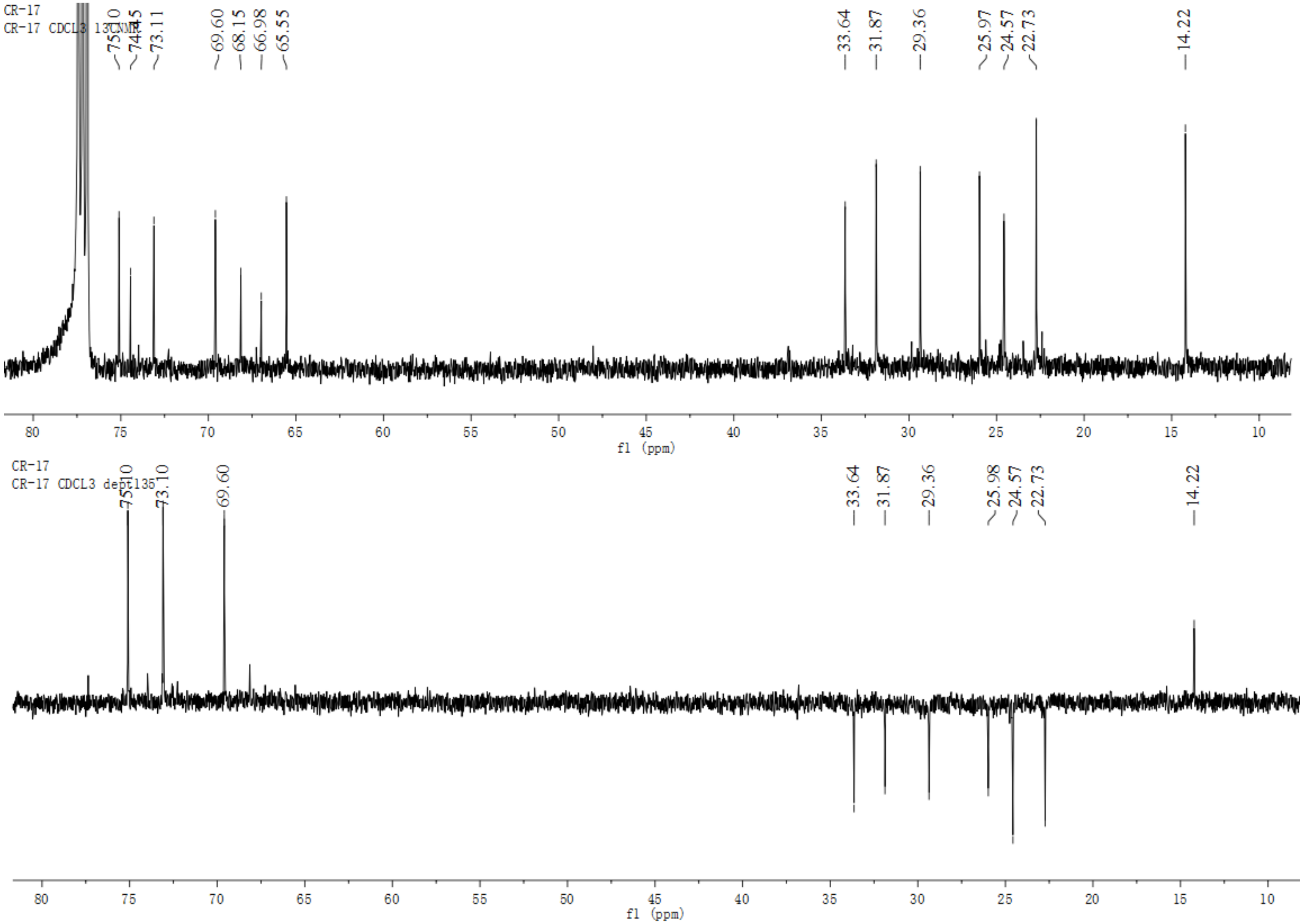

Figure S23. ^{13}C NMR spectrum (125 MHz) of compound **4** in $CDCl_3$.

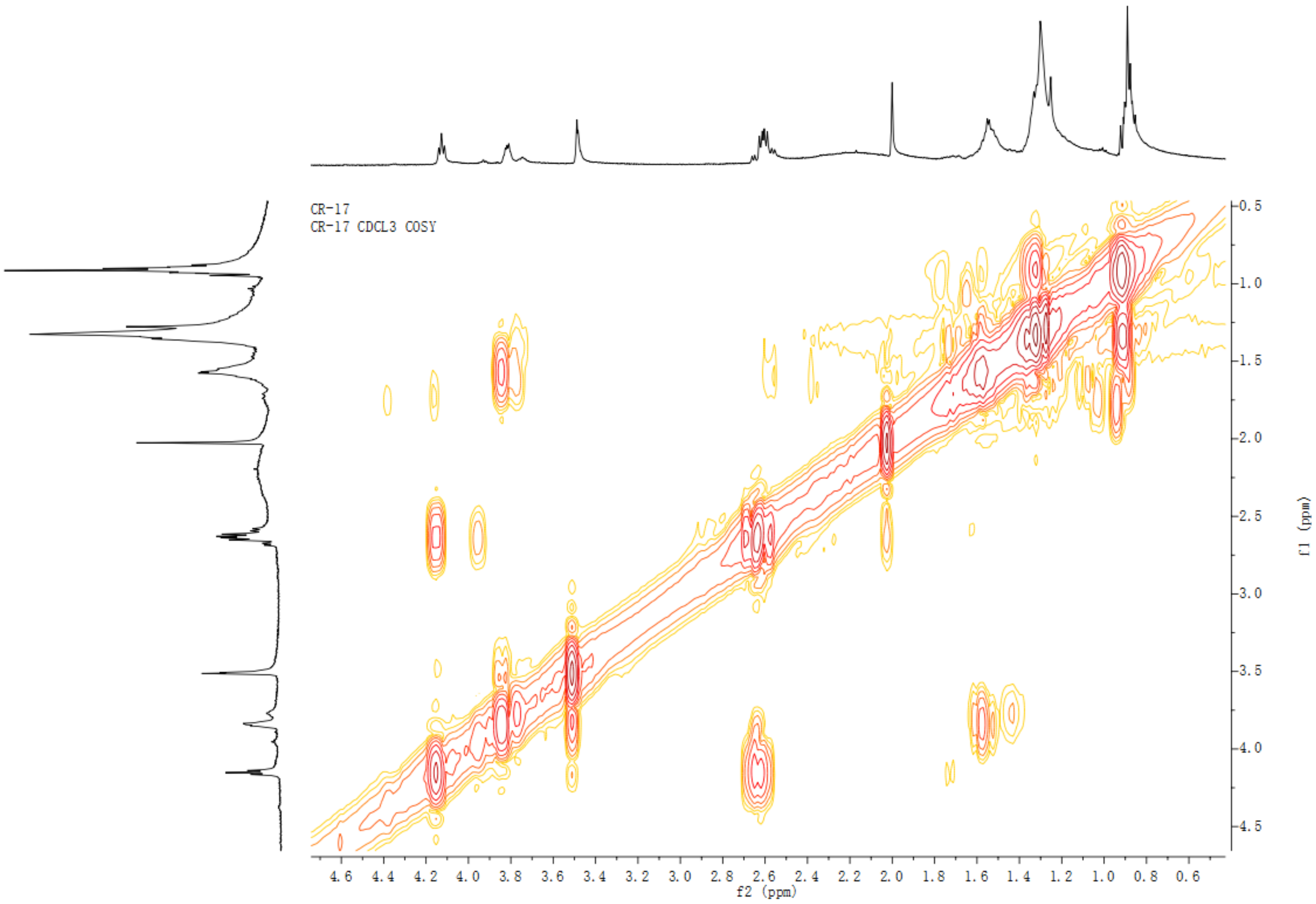

Figure S24. ^{1}H–^{1}H COSY spectrum of compound **4** in $CDCl_3$.

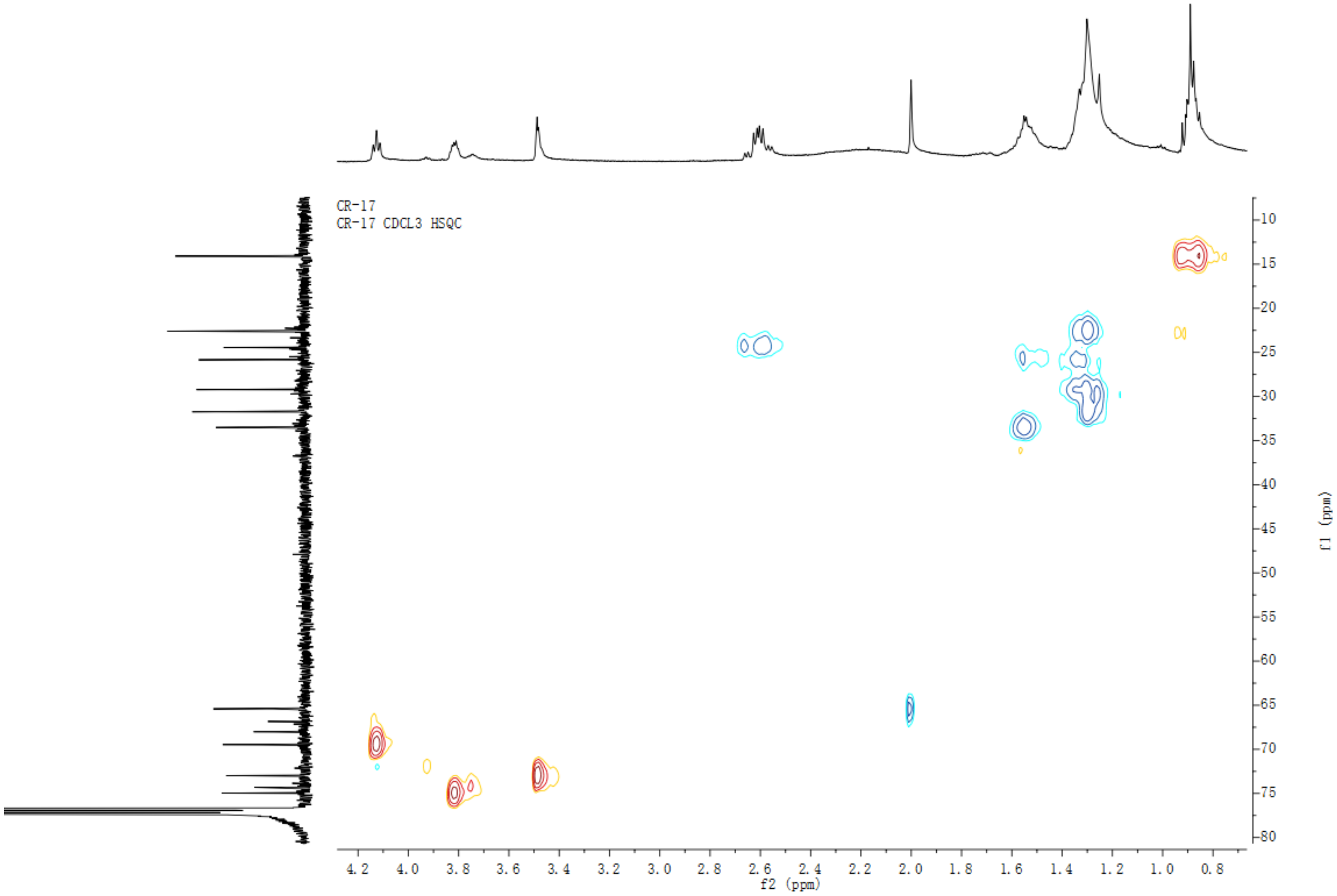

Figure S25. HSQC spectrum of compound **4** in $CDCl_3$.

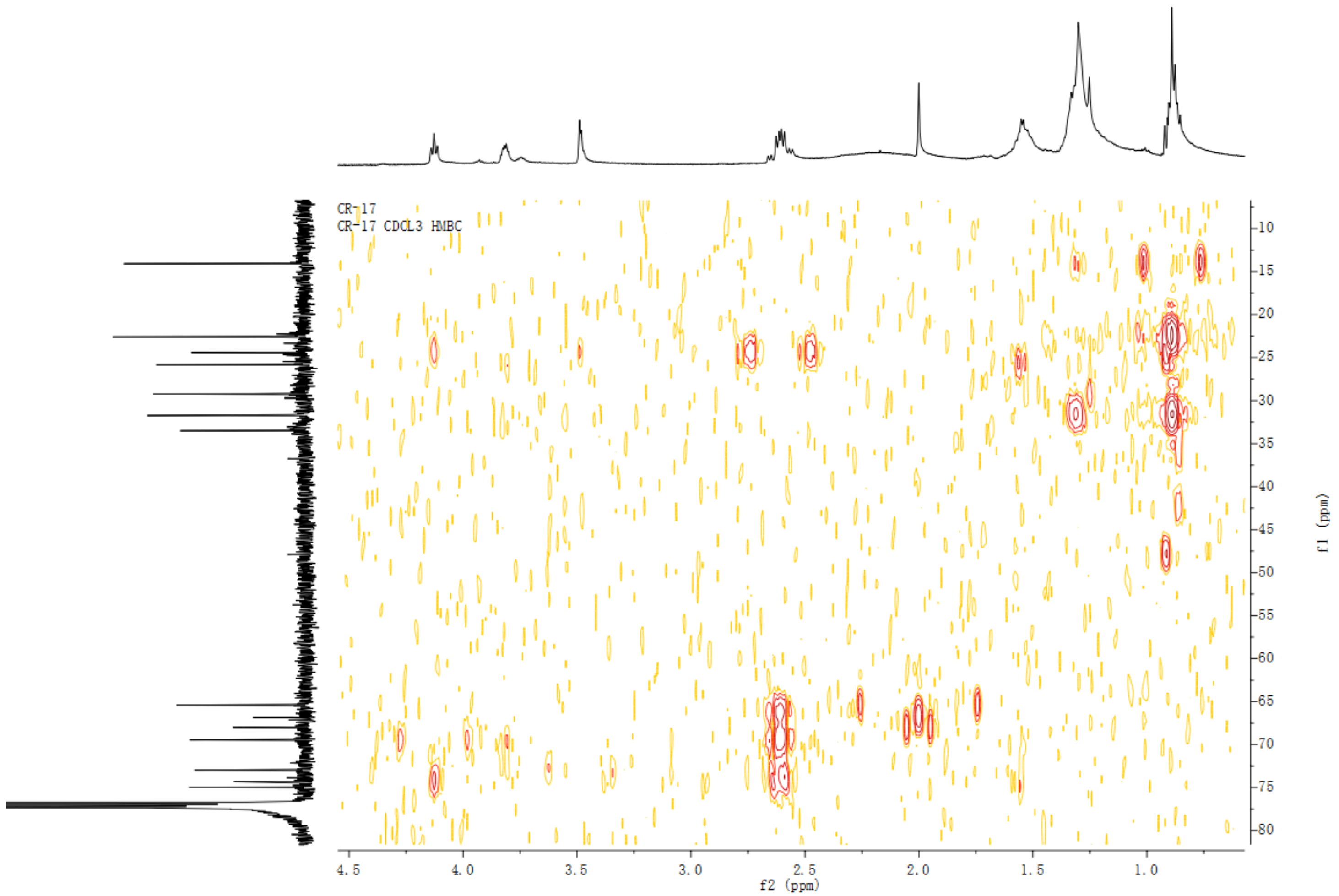

Figure S26. HMBC spectrum of compound **4** in $CDCl_3$.

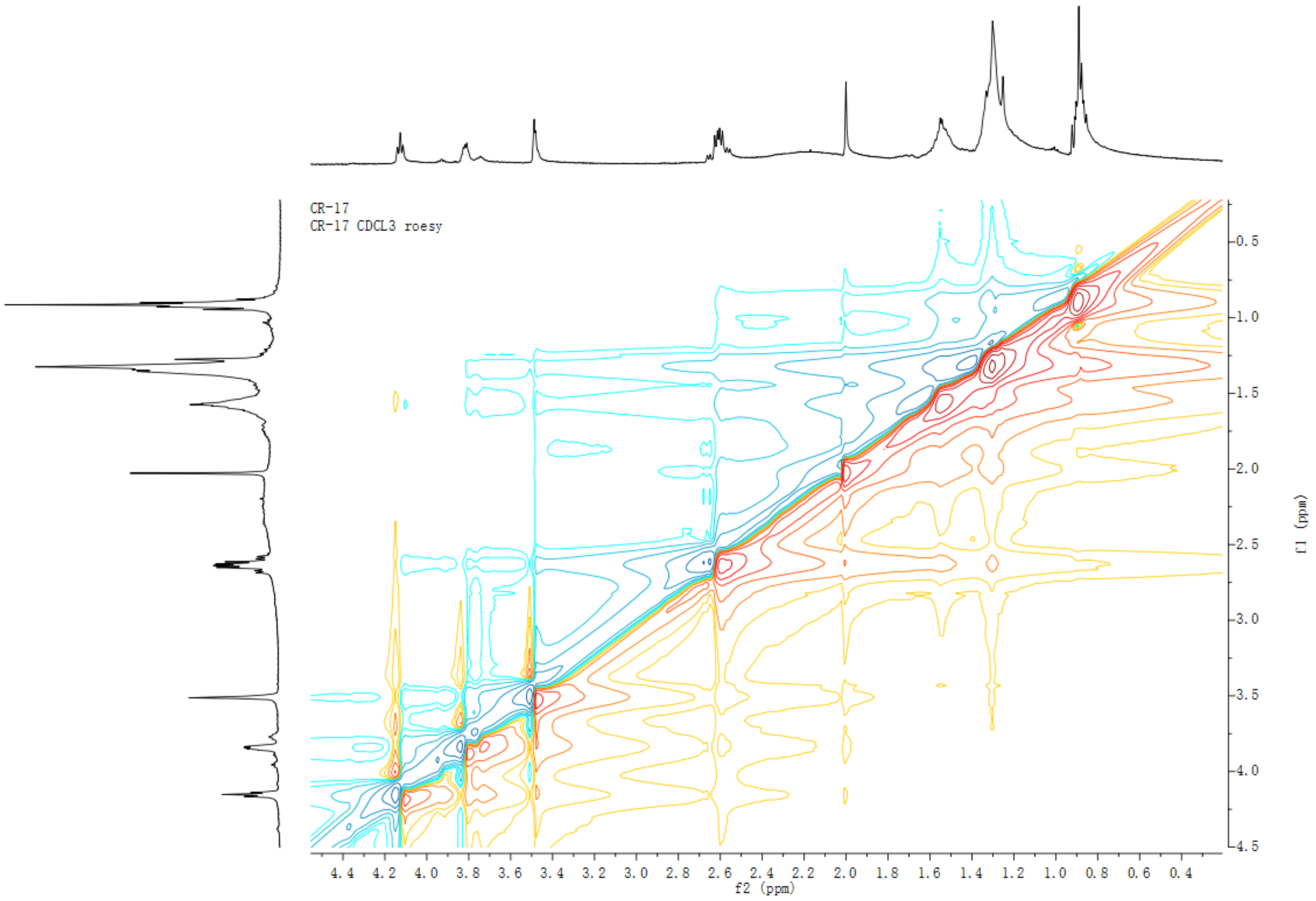

Figure S27. ROESY spectrum of compound **4** in $CDCl_3$.

Qualitative Analysis Report

**Data Filename**	150203ESIA2.d	**Sample Name**	CR-17
**Sample Type**	Sample	**Position**	
**Instrument Name**	Agilent G6230 TOF MS	**User Name**	KIB
**Acq Method**	ESI.m	**Acquired Time**	2/3/2015 11:07:25 AM
**IRM Calibration Status**	Success	**DA Method**	ESI.m
**Comment**			

**Sample Group**		**Info.**
**Acquisition SW**	6200 series TOF/6500 series	
**Version**	Q-TOF B.05.01 (B5125.2)	

User Spectra

Fragmentor Voltage	Collision Energy	Ionization Mode
200	0	ESI

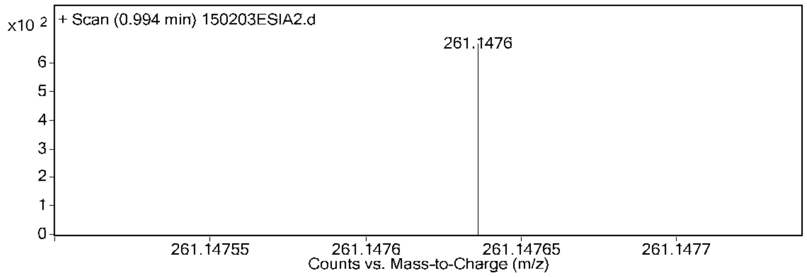

Peak List

m/z	z	Abund
121.0509	1	219630.8
122.0528	1	12863.49
135.0662		6055.44
274.2737	1	10609.1
318.2994	1	8079.25
922.0098	1	324422.59
923.0119	1	54759.75
924.0141	1	6479.02
1821.9503	1	17132.29
1941.9912	1	7572.69

Formula Calculator Element Limits

Element	Min	Max
C	0	200
H	0	400
O	0	5
Na	1	1

Formula Calculator Results

Formula	CalculatedMass	CalculatedMz	Mz	Diff. (mDa)	Diff. (ppm)	DBE
C14 H22 Na O3	261.1467	261.1461	261.1476	-1.5	-5.8	3.5000

--- End Of Report ---

Figure S28. HRESI(+)MS spectrum of compound **4**.

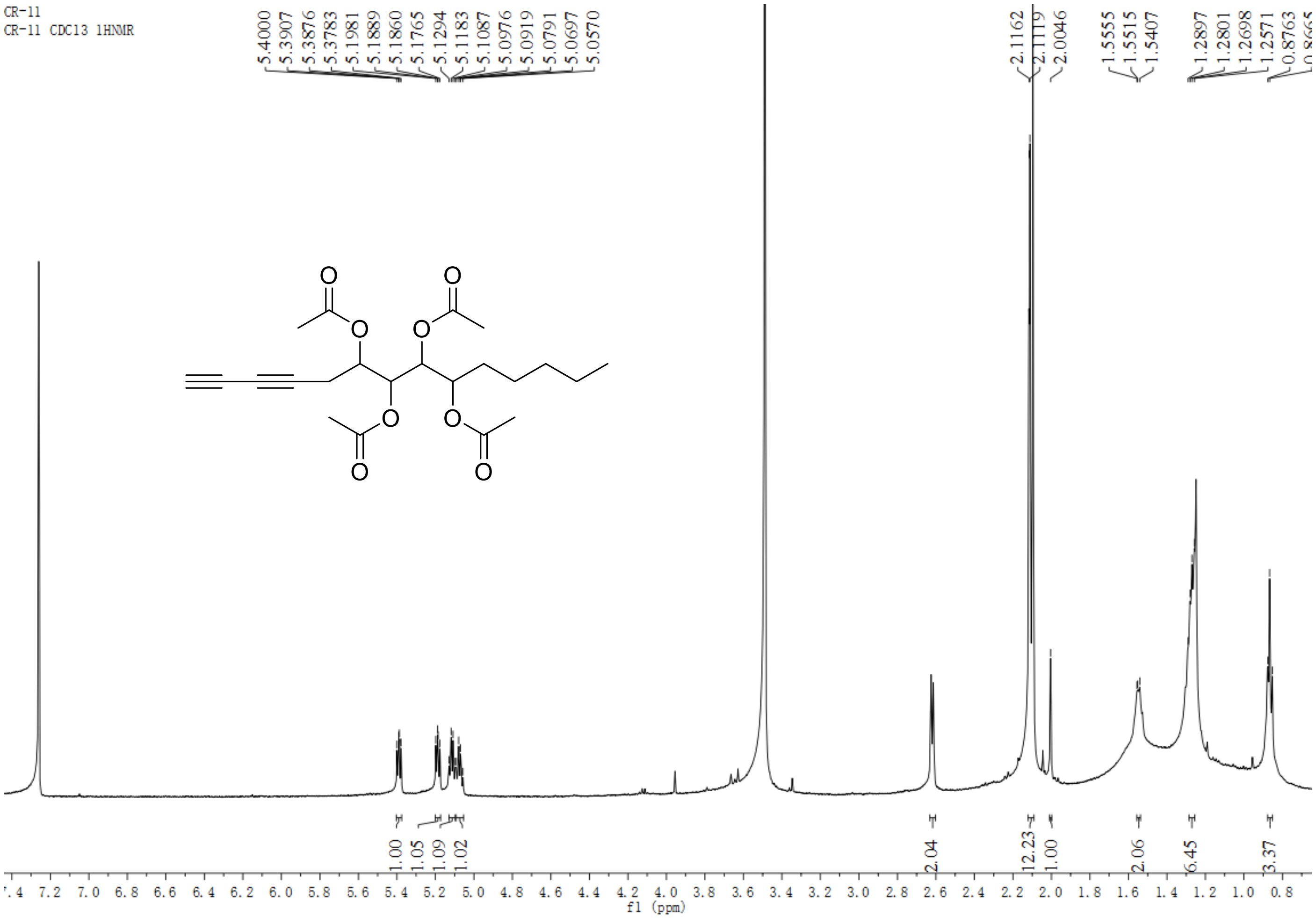

Figure S29. ^{1}H NMR spectrum (500 MHz) of compound **5** in $CDCl_3$.

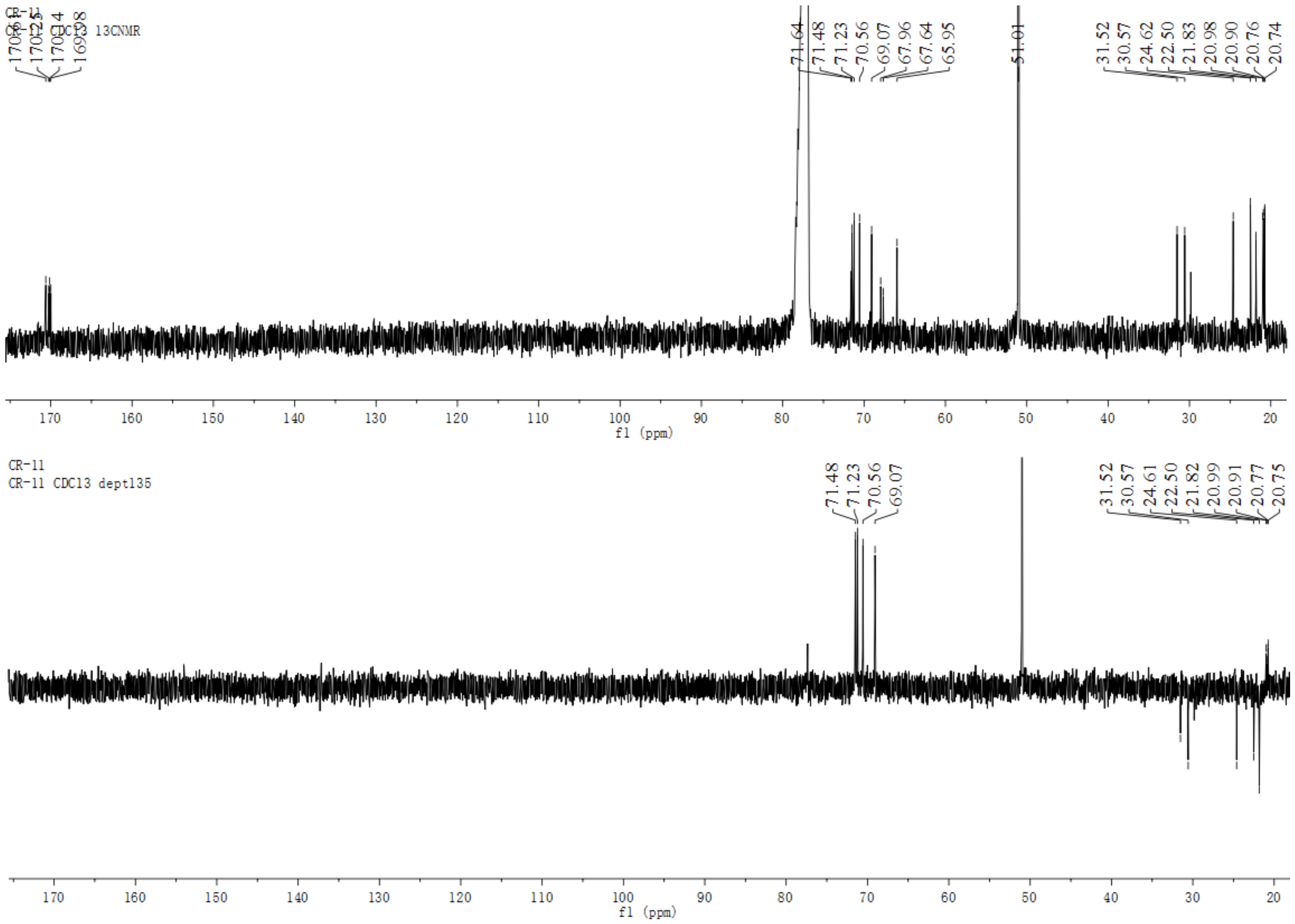

Figure S30. ^{13}C NMR spectrum (125 MHz) of compound **5** in $CDCl_3$.

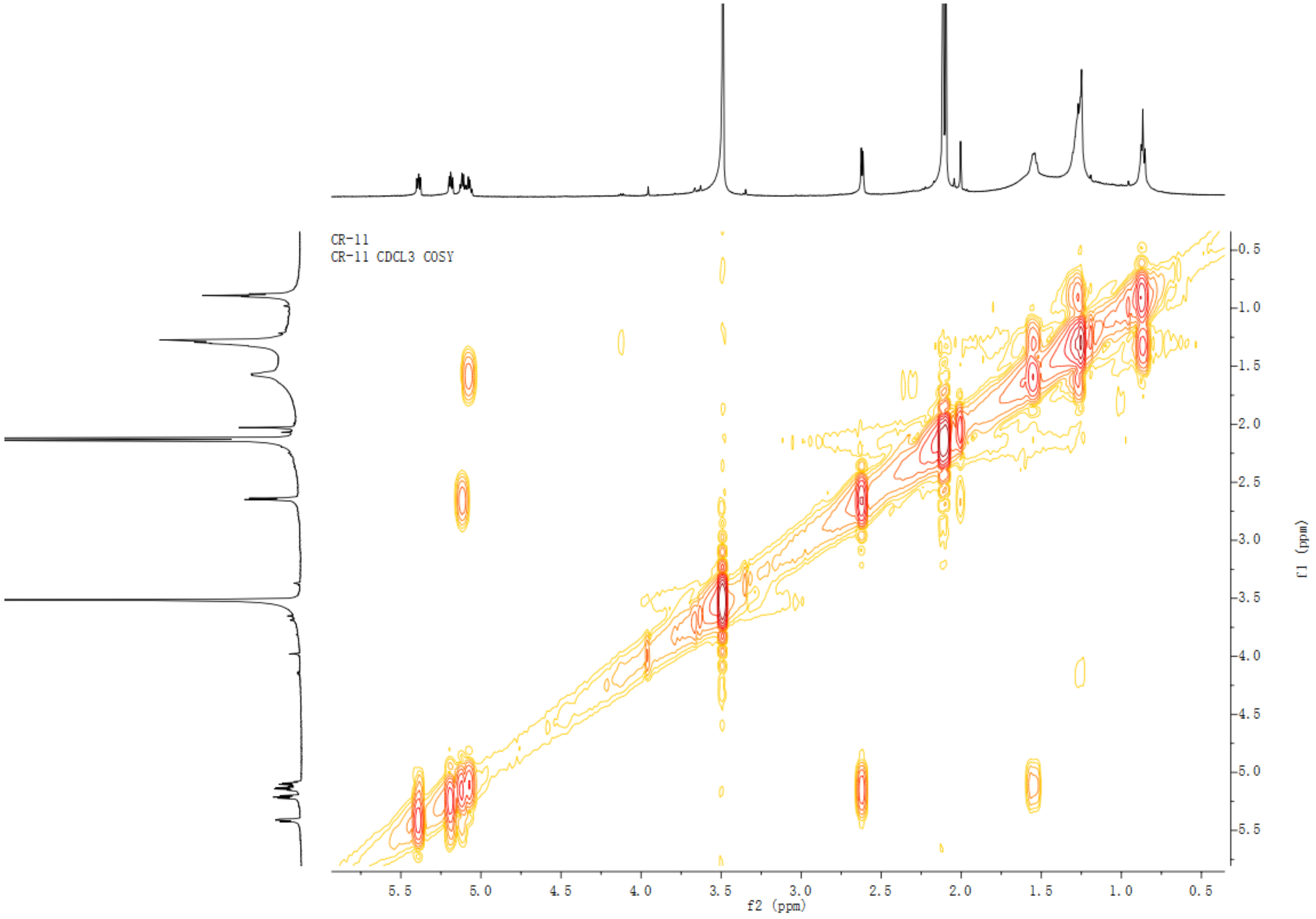

Figure S31. ^{1}H–^{1}H COSY spectrum of compound **5** in $CDCl_3$.

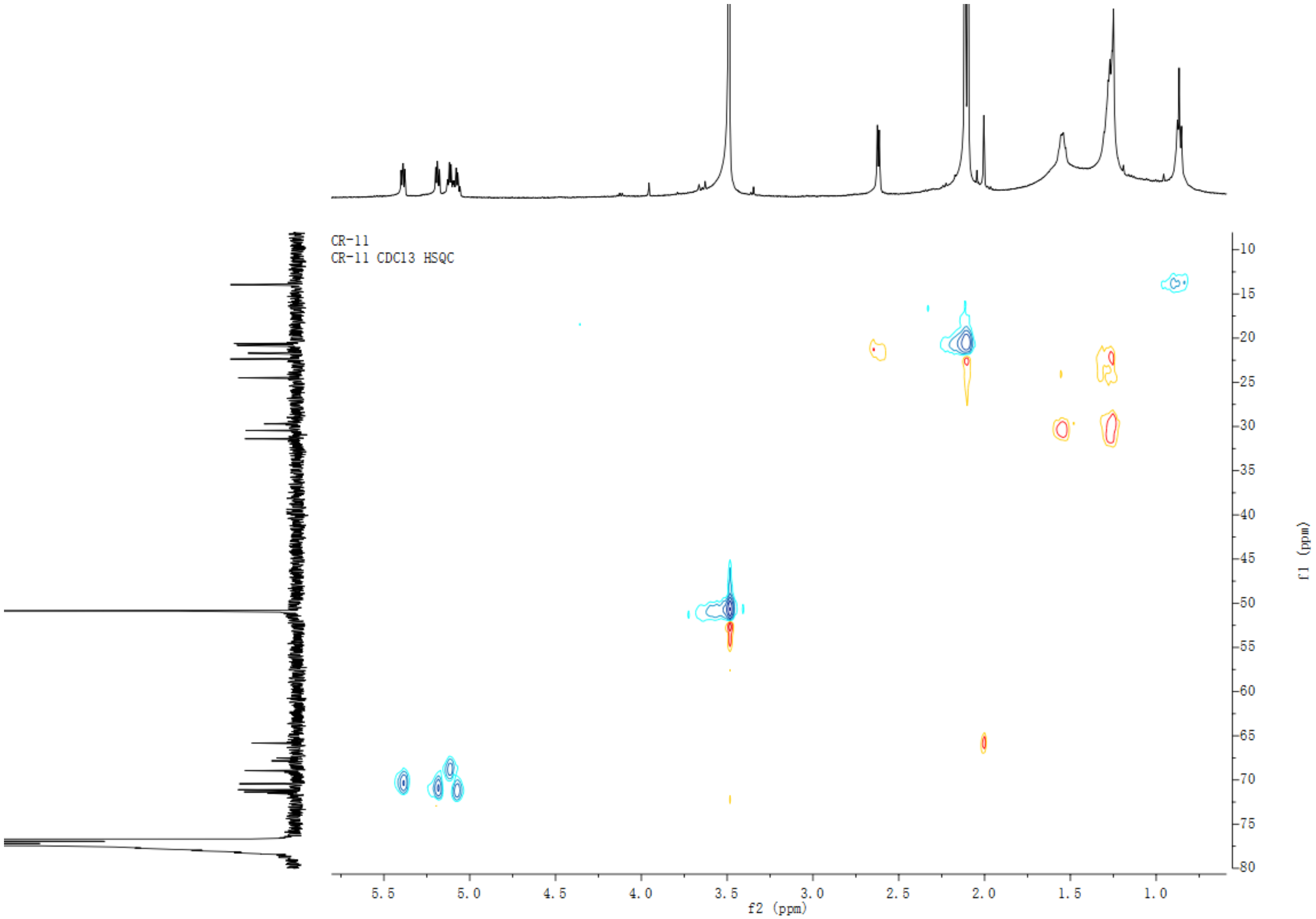

Figure S32. HSQC spectrum of compound **5** in $CDCl_3$.

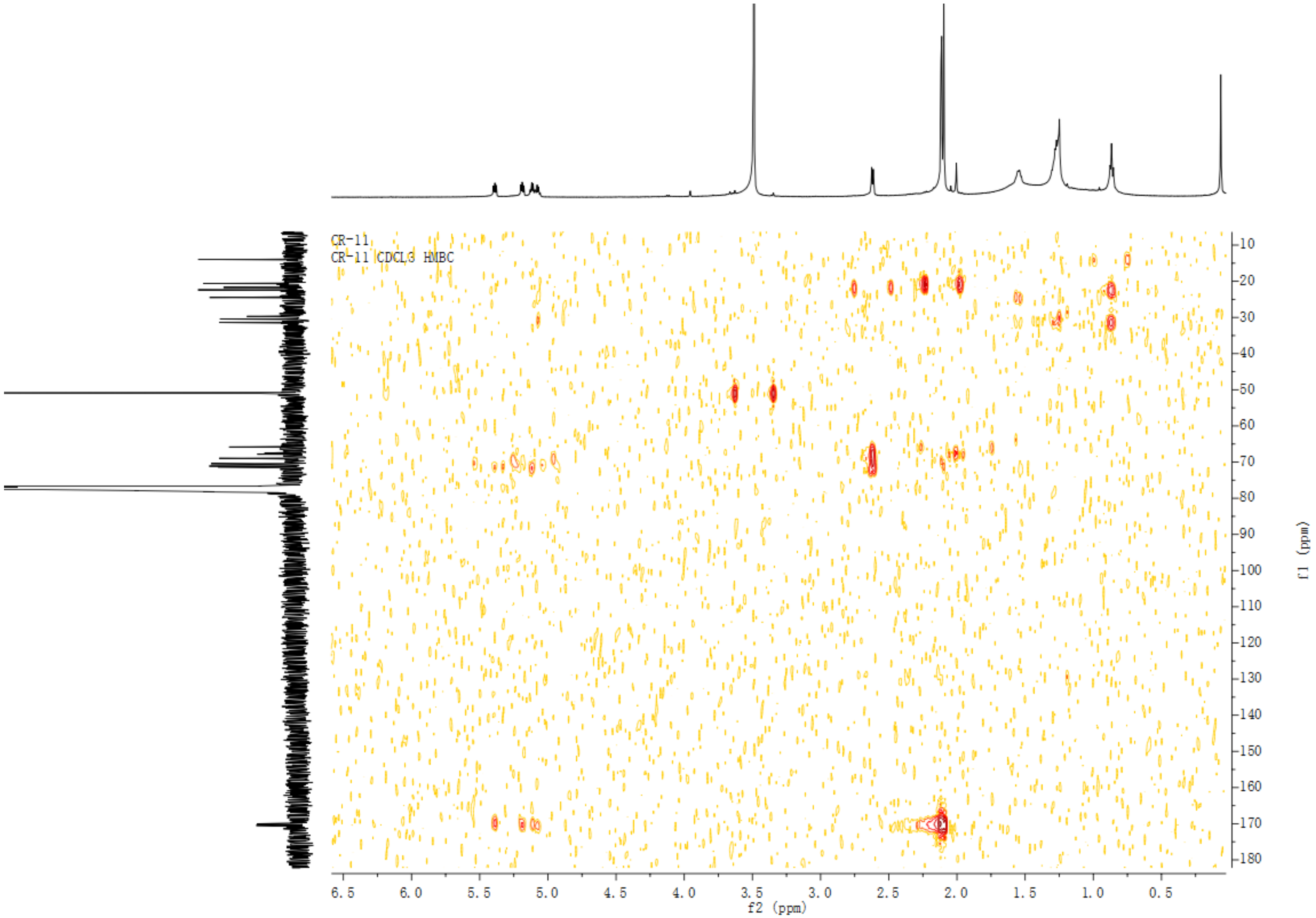

Figure S33. HMBC spectrum of compound **5** in $CDCl_3$.

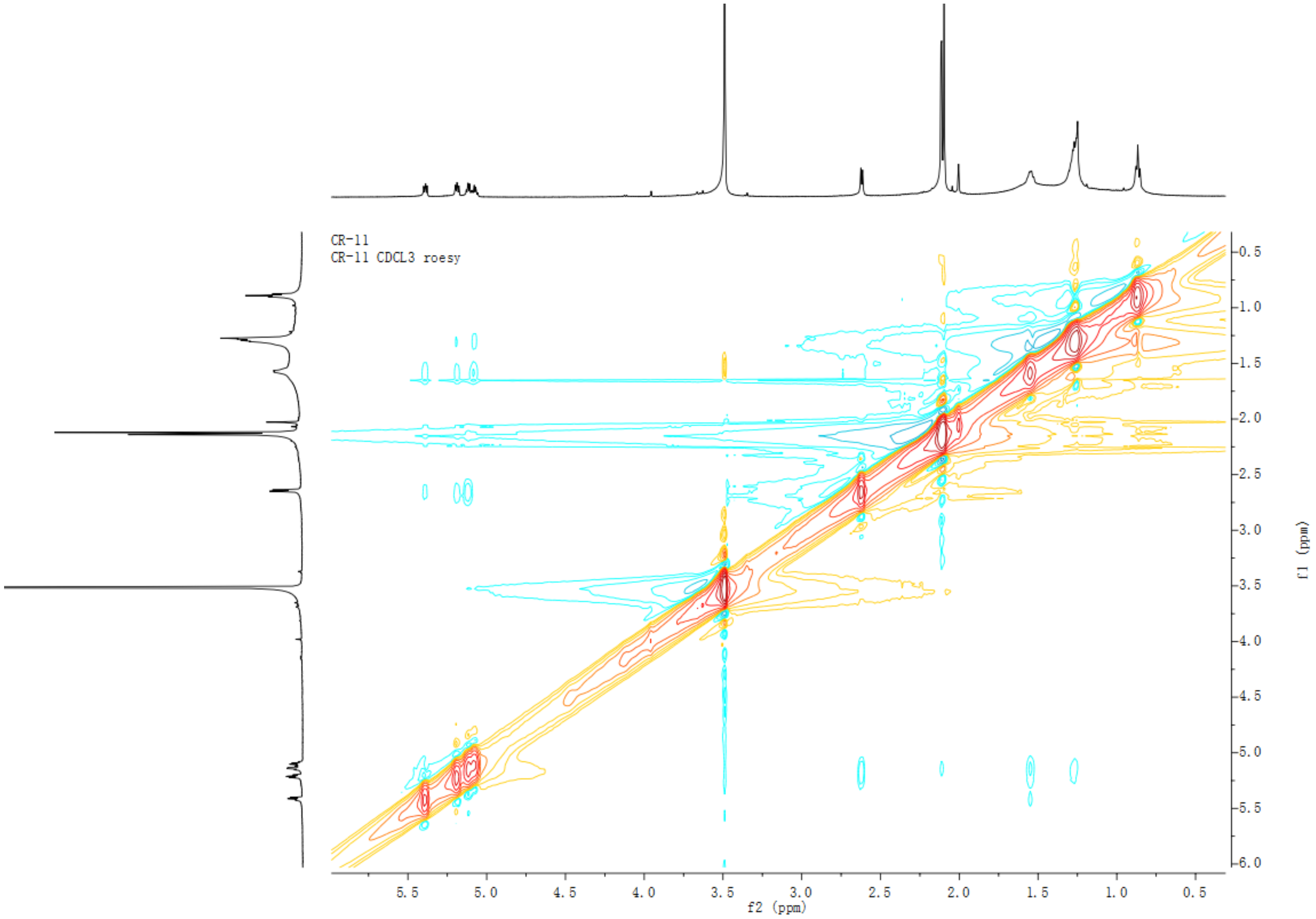

Figure S34. ROESY spectrum of compound **5** in $CDCl_3$.

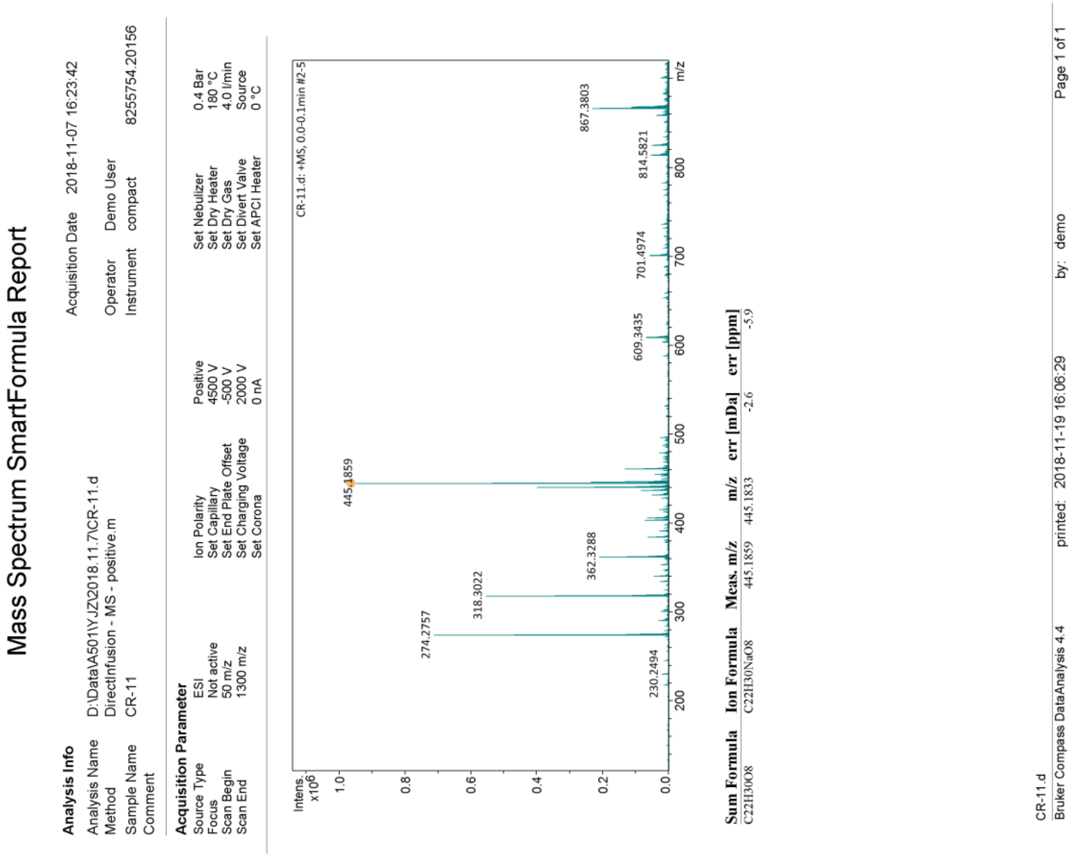

Figure S35. HRESI(+)MS spectrum of compound **5**.
